# A carotenoid-rich functional tomato sauce (OsteoCol®) reduces liver fat content in adults with metabolic dysfunction-associated steatotic liver disease: a randomized clinical trial

**DOI:** 10.1007/s00394-026-04049-w

**Published:** 2026-07-03

**Authors:** Rosario Mare, Yvelise Ferro, Samantha Maurotti, Elisa Mazza, Alberto Castagna, Carmelo Pujia, Francesca Rita Noto, Angelo Galluccio, Angela Mirarchi, Giuseppe Andrea Giampà, Marta Moraca, Tiziana Montalcini, Arturo Pujia

**Affiliations:** 1 Department of Medical and Surgical Sciences, University Magna Græcia, Catanzaro, Italy; 2 Department of Clinical and Experimental Medicine, University Magna Græcia, Catanzaro, Italy; 3 Clinical Nutrition Unit, AOU “Renato Dulbecco”, Catanzaro, Italy; 4 Research Center for the Prevention and Treatment of Metabolic Diseases, University Magna Græcia, Catanzaro, Italy

**Keywords:** Functional foods, Lycopene, Hepatic steatosis, Tomato sauce, Metabolic dysfunction-associated steatotic liver disease

## Abstract

**Background:**

Metabolic Dysfunction-Associated Steatotic Liver Disease (MASLD) is a common condition characterized by liver fat accumulation, and it has become a leading cause of liver-related morbidity and mortality worldwide. Currently, no drugs are approved to prevent or treat this condition. Carotenoids, especially lycopene, a potent antioxidant present in tomatoes, have demonstrated potential benefits for liver health in both preclinical and clinical studies. However, the lycopene content differs between tomatoes according to maturity stage. Ripening is an agronomic technique that enhances lycopene content by allowing tomatoes to fully mature on the plant. This study aimed to assess the effect of a carotenoid-rich tomato sauce made from vine-ripened tomatoes (OsteoCol®, patent N°:102019000000061 on liver fat in adults with MASLD.

**Methods:**

A total of 98 patients were enrolled in a double-blind, placebo-controlled clinical trial. The protocol was approved by the local Ethics Committee (124/2022/CE). The intervention group consumed the carotenoid-rich tomato sauce (OsteoCol®), while the control group received a commercial tomato sauce as a placebo for a total of 12 weeks.

The main outcome was the variation in hepatic fat content (CAP score). CAP score, by transient elastography, serum glucose, lipids, and transaminases were measured at baseline and after treatment. We also investigated the effect of OsteoCol® extract on rat hepatoma cells and human hepatocellular carcinoma cell lines.

**Results:**

A greater reduction in liver fat content was observed in participants consuming OsteoCol® compared to those receiving the placebo (CAP score: − 33 ± 44 dB/m vs. − 12 ± 38 dB/m, *p* = 0.014). The percentage reduction in the CAP score was even greater in men, individuals aged 60 and older, those with normal weight/overweight, and adults with mild hepatic steatosis at baseline. Additionally, in vitro studies on hepatic cell lines supported the clinical findings by demonstrating that OsteoCol® extract significantly reduced intracellular lipid accumulation through modulation of lipogenesis, β-oxidation, and cholesterol synthesis pathways.

**Conclusions:**

A functional tomato sauce made from vine-ripened tomatoes reduced liver fat content in adults with MASLD. This carotenoid-rich tomato sauce could serve as a novel nutritional strategy for patients with MASLD.

*Trial registration* ISRCTN, ISRCTN10483954. Registered 04 June 2025—retrospectively registered, https://doi.org/10.1186/ISRCTN10483954

**Supplementary Information:**

The online version contains supplementary material available at 10.1007/s00394-026-04049-w.

## Introduction

Metabolic dysfunction-associated steatotic liver disease (MASLD) is a globally prevalent liver condition, strongly associated with metabolic factors including insulin resistance, type 2 diabetes, and obesity [1]. The worldwide prevalence of MASLD among adults is estimated to be approximately 38%, while in Europe, it reaches as high as 25% of the adult population [2]. However, this percentage can rise to 50% in the obese population [3] and reach almost 60% in people with type 2 diabetes mellitus (T2DM). By 2040, the prevalence of MASLD among adults is expected to rise beyond 55% [4]. MASLD is a progressive condition that can progress from isolated fatty liver disease to metabolically dysfunction-associated steatohepatitis (MASH), with varying degrees of liver fibrosis that can progress to cirrhosis, liver failure, and hepatocellular carcinoma (HCC) [1, 2]. Besides liver-related complications, MASLD is associated with an increased risk of extrahepatic manifestations, including certain types of cancers, chronic kidney and cardiovascular disease [2]. Furthermore, MASLD is associated with an increased risk of impaired quality of life, disability, mortality, and healthcare costs [2, 4, 5]. This underlines the need to find strategies for the prevention and treatment of this disease. Due to the strong association between MASLD, obesity and related metabolic diseases, lifestyle interventions focused on body weight reduction (5–10%) through dietary modifications and physical exercise represent key strategies in MASLD management [1]. However, a substantial percentage of individuals with MASLD do not adhere to this effective treatment regimen [1, 6]. While, there are currently no drugs approved to prevent and treat MASLD, recent advances include the Food and Drug Administration approval and a positive opinion from European Medicines Agency for resmetirom for the treatment of MASH with fibrosis [7].

Additionally, glucagon-like peptide-1 receptor (GLP-1R) agonists and tirzepatide have demonstrated promising effects on liver fat reduction and resolution of MASH in clinical trials [8, 9], though they are not yet approved for this indication.

Some naturally occurring bioactive compounds extracted from fruits and vegetables appear to have beneficial effects in the treatment of MASLD [10–13]. Tomato and tomato-based products are important dietary sources of carotenoids, with lycopene representing the predominant carotenoid and β-carotene also contributing to their carotenoid profile [14]. In the European population, daily lycopene consumption typically was between 0.5 and 5 mg [15].

However, the amount of carotenoids in tomato products, and in particular in commercial tomato sauces [16], can vary significantly due to factors such as cultivar differences, growing conditions, maturity stage, storage temperatures of the tomatoes and processing technologies [17–20]. Additionally, tomatoes that ripen on the vine contain significantly higher levels of lycopene and other carotenoids than those that ripen after being picked [21, 22]. The beneficial health effects of lycopene are mainly attributed to its antioxidant and anti-inflammatory action [14, 23–25]. Additionally, several preclinical and clinical studies have demonstrated beneficial effects of lycopene on fat accumulation in the liver [26–29]. Furthermore, epidemiological studies demonstrate that a higher dietary intake of carotenoids is associated with a lower risk of having MASLD [30–32]. Finally, a recent study showed that consuming 9.50–10.00 mg/day of lycopene extracts from cooked and fresh tomatoes is effective in reducing the risk of hepatic fat accumulation in adults, suggesting a potential protective effect [33]. However, despite this evidence, to date, there is a notable lack of clinical studies on the potential beneficial effects of consuming carotenoids contained in tomatoes and derived products in the prevention and treatment of MASLD. OsteoCol® is a patented, commercially available functional tomato sauce naturally enriched with carotenoids, made from vine-ripened tomatoes of a local organic variety in southern Italy. In previous studies, we have shown that OsteoCol® tomato sauce contains a higher amount of lycopene than to other commercially available tomato sauces [16], and that it reduces serum cholesterol levels in adults with polygenic hypercholesterolemia [34]. Therefore, based on existing literature, we hypothesized that this carotenoid rich functional tomato sauce might also have beneficial effects on MASLD. To explore this, we conducted a study to evaluate the effects of OsteoCol® as a treatment for adults with MASLD. Additionally, we investigated whether the carotenoid extract from this tomato sauce could reduce intracellular lipid content in cellular models of hepatic steatosis.

## Methods

### Human study

This parallel randomized, double-blind, placebo-controlled clinical trial (RCT) was conducted from March 12, 2023, to August 23, 2023. The study included adults of both sexs, aged 30–75 years, with a diagnosis of with MASLD who were attending the Clinical Nutrition Unit of the “Renato Dulbecco” University Hospital of Catanzaro, Italy. MASLD is defined by the presence of excess triglyceride storage in the liver, as assessed by Transient Vibration-Controlled Elastography (VCTE), along with at least one cardiometabolic risk factor [1]. In particular, excess triglyceride accumulation in the liver is defined by for a Controlled Attenuation Parameter value (CAP) ≥ 247 dB/m (34). The RCT protocol was approved by the Territorial Ethics Committee of the Calabria Region (124/2022/CE April 21nd, 2022). This RCT was registered in the International Clinical Trials Registry Platform (ICTRP) with the identifier ISRCTN10483954. For this study, we excluded individuals with allergy to tomato and nickel or a diagnosis of chronic hepatitis B and/or C virus infection. According to the protocol, we excluded subjects with triglycerides concentration over 250 mg/dl, T2DM, autoimmune or cholestatic liver disease, liver cirrhosis, gastroesophageal reflux, nephrotic syndrome, pregnancy, chronic renal failure, and affected by debilitating diseases. Furthermore, adults with previous and actual alcohol abuse (> 20 g of alcohol per day) [1], in treatment with methotrexate, corticosteroids, amiodarone, antiretroviral agents, valproate, tamoxifen, nutraceuticals, supplements or functional foods to reduce excess fat in the liver as confirmed by their medical records.

#### Study design

Participants are allocated with a simple randomization using computer-generated random numbers to the intervention or the control group. Ninety-eight adults were randomly assigned to two groups for 12 weeks, as follows:

Intervention group: Participants received a functional tomato sauce (OsteoCol®), naturally enriched with carotenoids, produced from vine-ripened, organic Calabrian tomatoes, harvested at full physiological maturity directly on the plant. Twenty-five participants consumed 80 g/day, while twenty-four participants consumed 160 g every other day.

Control group: Participants received a commercial tomato sauce made from non-organic tomatoes. In this case, no vine-ripening procedure was applied, as the tomatoes were harvested before reaching full maturation on the plant, in line with typical industrial processing methods. Twenty-five participants consumed 80 g/day, while twenty-four participants consumed 160 g every other day.

The two different administration regimens (80 g/day or 160 g every other day) were designed exclusively to facilitate patient adherence and accommodate individual dietary preferences. Since the primary objective of this study was to evaluate the overall clinical efficacy of functional tomato sauce on liver fat content regardless of the daily schedule or frequency of administration, participants in each macro-intervention arm were pooled for the primary statistical analyses, in line with statistical power calculations.

Moreover, participants were explicitly instructed to consume the tomato sauce cooked rather than raw. To maximize dietary compliance and standardize the intervention, all subjects received a standardized recipe booklet containing various options (e.g., pasta dishes, fish, or meat preparations). All recommended recipes included the addition of olive oil.

Both tomato sauces utilized in this RCT were supplied by C.G. Food SRL, Soverato, Italy. Nutritional profile of both sauces is listed in Supplemental Table 1.

According to the protocol, each participant received 10 bottles of 700 ml of tomato sauce, provided by a registered dietician based on the study arm allocation.

The primary outcome measured was a change in the amount of intrahepatic fat, assessed by CAP score, after a 12-week intervention between groups.

In this RCT, both the researchers and participants were blinded to treatment allocation. All participants signed written informed consent. The investigation conforms to the ethical principles outlined in the Declaration of Helsinki.

#### Dietary intake and adherence to treatment

In this RCT, we evaluated dietary habits using a 71-items food frequency questionnaire (FFQ) used in previous studies [35] at baseline and after 12 weeks. All participants were given oral and written instructions to follow the Mediterranean Diet recommendations to promote healthy eating habits [36] without any energy restriction. A registered dietician provided dietary advice and treatments to both groups. Each patient receives printed copy of these dietary recommendations [36]. To monitor intake of functional or control tomato sauce, participants were given with an adherence diary to record each day they consumed the product. These diaries were given back to the research team to evaluate adherence compliance. Furthermore, they were asked to collect and return the bottle caps as a further measure of adherence. At the end of the RCT, participants were considered adherent if they had consumed at least 80% of the provided tomato sauce.

#### Anthropometric measurements and cardiovascular risk factors assessment

Body weight was assessed using a calibrated digital scale (Tanita DC-430 MA) with a precision of 0.1 kg, while height was determined with a stadiometer (seca 213) with an accuracy of 0.1 cm. Body mass index (BMI) was calculated by dividing weight in kilograms by the square of height in meters. Waist circumferences (WC) and hip circumferences (HC) were measured with a non-stretchable tape measure. WC was assessed over the unclothed abdomen at the narrowest point between the costal margin and the iliac crest. HC was measured over light clothing at the level of the widest diameter around the buttocks. To evaluate fat distribution, we utilized measurements of WC and HC to calculate the waist-to-hip ratio (WHR) [36]. Systemic blood pressure was measured at baseline and follow-up visits. We also assessed the presence of the cardiometabolic risk factors (i.e. dyslipidemia and hypertension), and smoking habit, from participant interview and medical records [36]. Additionally, all patients were evaluated for the presence of metabolic syndrome (MetS) [37].

#### Physical activity assessment

Baseline physical activity levels of the participants were quantified using the validated short form of the Neighborhood Physical Activity Questionnaire (NPAQ-short) [38]. Based on the recorded scores, subjects were categorized into two distinct cohorts: those meeting the criteria for moderate-to-vigorous physical activity (MVPA) and those demonstrating sedentary habits or light physical exertion. The MVPA category encompassed activities such as recreational sports, brisk walking, dancing, gardening, and walking domestic pets [39].

#### Intrahepatic fat assessment

The amount of hepatic fat (CAP score) and liver stiffness measurement (LSM) were measured using VCTE device (Fibroscan® 630, Echosens™, Paris, France) by a single experienced operator who was blinded to both the patient’s treatment allocation and clinical data. All measurements were performed using a 3.5 MHz standard M probe on the right hepatic lobe through the intercostal spaces with the patient lying supine position, as previous described [12, 36]. Measurements were considered valid if they included 10 successful acquisitions, and achieved a success rate of at least 60%. Only measurements meeting these criteria were included in the study. The final LSM and CAP score were recorded as the median values of all measurements, and they were expressed in kPa and dB/m, respectively. The interquartile range (IQR) was used to assess the variability of LSMs, while the standard deviation (SD) was used to assess the variability of CAP scores. An IQR/median ratio < 30% and a CAP SD < 40 dB/m were considered indicators of reliable measurements. The diagnosis of liver fibrosis was established for a cut-off value of LSM > 7 kPa. The CAP score having ranging between 100 and 400 dB/m, and diagnosis of excess hepatic triglyceride accumulation was based on a CAP score ≥ 247 dB/m [36]. Thus, all participants were categorized into three grades of hepatic steatosis severity: Mild (S1) with a CAP score between 247 and 268 dB/m, moderate (S2) with a CAP score between 269 and 280 dB/m, and severe (S3) with a CAP score ≥ 281 dB/m [12, 36]. All scans were performed by the same operator.

#### Biochemical evaluation

Venous blood was collected after fasting overnight into vacutainer tubes (Becton & Dickinson, Plymouth, England) and centrifuged within 4 h. At baseline and after 12 weeks, using a chemiluminescent immunoassay according to the manufacturer’s instructions on a COBAS 8000 (Roche, Switzerland), we measured serum insulin, glucose, creatinine, total cholesterol (TC), high density lipoprotein cholesterol (HDL-C), triglycerides (TG), alanine aminotransferase (ALT), aspartate aminotransferase (AST), γ-glutamyltransferase (γGT), albumin, and c-reactive protein (CRP). Friedewald formula was used to calculate low-density lipoprotein cholesterol (LDL-C) [40]. We also calculated the homeostatic model assessment (HOMA) index [41].

#### Safety measurements and adverse events

We evaluated various parameters related to overall health, including systemic blood pressure, as well as serum concentrations of creatinine, transaminases, lipids, and glucose. To document adverse events (AEs), we utilized a patient-reported outcome questionnaire. This questionnaire was administered to monitor any new symptoms emerging after enrolment in the RCT that could potentially be associated with the nutritional treatment. Additionally, the questionnaire enabled assessment of the type and severity of the reported adverse events.

### In vitro study

#### Carotenoids content analysis

Carotenoids were extracted from both tomato sauces according to a previously established method [1]. Briefly, both tomato sauces were incubated with hexane (1:2 v/v) under continuous stirring (400 rpm) for 2 h, followed by centrifugation. The supernatant was filtered (0.45 µm nylon filters) and stored at − 20 °C, protected from light. The extracted carotenoids were quantified by UV–Vis spectrophotometry and high-performance liquid chromatography (HPLC). The UV–Vis measurements were performed with a ThermoFisher Scientific Genesys150™ spectrophotometer equipped with quartz cuvettes (1 cm path length) and maximum absorbance (AbsMAX) was determined in the range of 300–650 nm. HPLC analysis was performed on a ThermoFisher Scientific Vanquish system equipped with a C18 reverse-phase Acclaim® 120 column (100 mm × 4.6 mm, 5 µm). The mobile phase was methanol/acetonitrile (8:2 v/v) at a flow rate of 1.2 mL/min and detector was set at 457 nm and 478 nm. Identification peaks were based on retention times relative to lycopene and β-carotene standards. Calibration curves (10–1000 ppm) were used for carotenoid quantification in 10 µL sample.

The antioxidant power and free radicals scavenging activity of carotenoids contained in sauces were investigated according to a protocol previously described in the literature based on the incubation with 2,2-Diphenyl-1-picrylhydrazyl (DPPH) [42]. For the assay, samples (25 μL) were incubated with 0.004% w/v radical solution. The absorbance was detected at wavelength (λ) = 517 nm after 30 min incubation in the dark. The variation in radical concentration was detected and quantified by a UV–Vis spectrophotometer, and the percentage of inhibition was calculated using the following equation:

$$ {\mathrm{I}}\left( \% \right) = \left[ {\left( {{\mathrm{A}}_{0} - {\mathrm{A}}_{{1}} } \right)/{\mathrm{A}}_{0} } \right] \times {1}00 $$

A_0_ = absorbance of negative control; A_1_ = absorbance of extracts/standards.

### In vitro digestion protocol

The in vitro gastrointestinal digestion of the Osteocol® sauce was simulated using a bench-top bioreactor (Applikon® MiniBio, Applikon Biotechnology, The Netherlands), regulated by a my-Control system to monitor and maintain temperature, pH, stirring speed, O_2_ insufflation, and CO_2_ emission. Prior to digestion, the Osteocol® sauce underwent a cooking process at 350 °C for 1 h. The digestion protocol was adapted from the standardized INFOGEST static in vitro method [43], introducing minor adjustments to accommodate the composition of the semi-solid food matrix. Throughout the process, the matrix was incubated at a constant temperature of 37 °C under continuous stirring at 200 rpm. The sequential digestive stages were executed utilizing stock aqueous solutions containing Protease (82,000 U/L), Cellulase (3500 U/L), α-Amylase (28,000 U/L), α-Galactosidase (600 U/L), Maltase (32,000 U/L), Lactase (680 U/L), Lipase (2100 U/L), Invertase (150 U/L), and Phytase (1.7 U/L), supplemented with soy lecithin (100 mg/L) and a mixture of fatty acids and magnesium salts. The gastric compartment reached a final volume of 250 mL, which incorporated 100 g of the pre-cooked sauce. The simulated digestion comprised an oral phase lasting 5 min at a controlled pH of 4.0, followed by a gastric phase maintained for 60 min at pH 2.5, and a final intestinal phase lasting 60 min at pH 7.0. Adjustments to the pH between consecutive stages were achieved via the addition of 1 M hydrochloric acid (HCl) and 1 M sodium hydroxide (NaOH) solutions. To monitor the digestate at the transition points between the distinct gastrointestinal phases, 5 mL aliquots were sampled from the bioreactor. These fractions were immediately transferred to a refrigeration unit (temp. 4 °C) to halt enzymatic activity and subsequently stored at − 20 °C until downstream chemical characterization. Lycopene content within each digestive phase was extracted and quantified by UV–Vis Spectrophotometer and HPLC according to the procedure described above (Supplemental Table 2).

### Carotenoids micellar suspension for in vitro study

The solubility of carotenoids in aqueous medium was guaranteed by forming a surfactant-based micellar suspension. In detail, Tween80® was solubilized in milli-Q® water with a concentration between 0.5 and 1%w/v. Carotenoids extracted from functional tomato sauce with hexane completely dried by a rotary evaporator (Rotavapor–Buchi) equipped with a vacuum pump and solubilized again in acetone, thus obtaining the organic phase of the micellar suspension. The complete dissolution of carotenoids were ensured using a thermostated ultrasonic bath. The aqueous phase was maintained under continuous stirring (400 rpm) and 2 mL of acetone were added drop by drop. The formulation obtained was incubated overnight under gentle stirring (50 rpm). The evaporation of acetone led to the formation of Tween-coated micellar carotenoids suspended in water and suitable for in vitro study. The formation and quantification of micellar lycopene was confirmed by UV–Vis analyses with the onset of a new specific absorbance peak at wavelength ~ 350 nm.

### Cell culture

The rat hepatoma cells (McA-RH7777) and human hepatocellular carcinoma (HepG2) cell lines were procured from ATCC. McA-RH7777 cells were cultured in Dulbecco’s Modified Eagle Medium (DMEM) supplemented with 1 mM sodium pyruvate, 100 μg/ml streptomycin, 4 mM glutamine, and further enriched with 10% fetal bovine serum (FBS) in the presence of 5% CO_2_. HepG2 cells were cultured in Modified Eagle Medium (MEM) supplemented with 1 mM sodium pyruvate, 1 mM essential amino acids, 100 U/ml penicillin, 100 μg/ml streptomycin, 4 mM glutamine, and enriched with 10% FBS under 5% CO_2_ conditions. Both cell lines were harvested by trypsinization and sub-cultured twice weekly. Additionally, they were periodically tested for the presence of mycoplasma using the Mycoplasma PCR detection kit (G238, abm).

### Treatments

McA-RH7777 and HepG2 cells were treated with OsteoCol®, formulated as a carotenoid micellar suspension (CMS), at final concentrations of 0.1 μM and 0.5 μM for 24 h. Empty micelles were prepared using the same Tween 80®-based vehicle and the same final Tween 80® concentration used for the CMS, corresponding to 0.05% in the final treatment medium. Thus, empty micelles differed from OsteoCol® micelles only by the absence of the carotenoid extract and were used as vehicle controls in all in vitro experiments. All experimental conditions therefore contained the same final amount of Tween 80®. In parallel, to evaluate the contribution of lycopene alone to lipid accumulation, HepG2 cells were treated with isolated all-trans lycopene at final concentrations of 0.1 μM and 0.5 μM for 24 h, under the same experimental conditions applied for the OsteoCol® CMS.

### Cell viability

McA-RH7777 and HepG2 cells were seeded in 96-well plates at a density of 1 × 10^4^ cells per well. After a 24-h of treatments, cell viability was then evaluated using the MTT assay. Specifically, a 5 mg/mL MTT solution (Sigma-Aldrich, St. Louis, MO, USA) was added to each well and incubated at 37 °C for 4 h. Following incubation, the supernatant was discarded and replaced with 100 μL of dimethyl sulfoxide (DMSO) to solubilize the formazan crystals. The absorbance was measured at 570 nm using a microplate reader.

### Quantification of intracellular neutral lipid content

To evaluate intracellular lipid accumulation, McA-RH7777 and HepG2 cells were seeded on glass coverslips placed in 24-well plates at a density of 5 × 10^4^ and 10 × 10^4^ cells per well, respectively. Following treatment, cells were washed with phosphate-buffered saline (PBS) and fixed with 4% paraformaldehyde for 10 min at room temperature. Lipid droplets were visualized by staining with Oil Red O (Sigma-Aldrich, St. Louis, MO, USA) for 20 min, and nuclei were counterstained with DAPI (D9542-5MG; Sigma-Aldrich) for 10 min. All staining steps were performed at room temperature under light-protected conditions. Imaging was conducted using a Leica DM4 B Upright Microscope equipped with an HC FL PLAN 40x/0.65 objective (Corning, Kennebunk, USA). Quantitative image analysis was performed with ImageJ software (version 1.52 h, NIH), and the area stained with Oil Red O was normalized to the number of DAPI-positive nuclei.

### Western blotting

HepG2 cells were plated in 6-well dishes at a density of 3 × 10^5^ cells per well. Following treatment, the cells were lysed using Mammalian Protein Extraction Reagent (M-PER) from Pierce, Thermo Fisher Scientific. Western blot analysis of proteins from the total cell lysates was carried out following standard procedures. The following antibodies were utilized: Phospho-AMPKα (Thr172) (#50081, Cell Signaling); SREBP-2 (Phospho- Ser455) (A50871, Antibodies); Phospho-AKT1 (Ser473) (44-621G, Thermo Fisher); Phospho-mTOR (Ser2448) (#5536, Cell Signaling); SREBP1c (A88065, Antibodies); PPARγ (K.242.9, Invitrogen); PPARα (MAB3890, MILLIPORE); SIRT1 (H00023411-M01, AbNOVA); β-ACTIN (A5441, SIGMA-ALDRICH); ANTI-MOUSE (#1706516, BIORAD); ANTI-RABBIT (#1706515, BIORAD).

### HMG-CR inhibitory activity assay

The HMG-CoA Reductase Assay Kit (CS1090, Sigma-Aldrich, St. Louis, MO, USA) was employed following the manufacturer’s protocol. Absorbance at 340 nm was recorded over a 10-min period using a GLOMAX microplate reader (Promega, GM3030). The assay measured NADPH oxidation in the presence and absence of inhibitors, and enzyme inhibition was calculated using the formula:

$$ \begin{aligned} \% {\mathrm{HMG}} - {\mathrm{CoAR}}\;{\mathrm{Inhibition}} & = \left( {\left( {\Delta {\text{Abs 1}}00\% {\text{ activity}}} \right.} \right. \\ & \quad \left. {\left. { - \Delta {\text{Abs sample}}} \right)} \right)/\left( {\Delta {\text{Abs 1}}00\% {\text{ activity}}} \right) \\ & \quad \times {1}00 \\ \end{aligned} $$

$$ \begin{aligned} \% {\mathrm{Inhibition}} & = \left( {\left( {\Delta {\text{Abs 1}}00\% {\mathrm{activity}}} \right.} \right. \\ & \quad \left. { - \Delta {\text{Abs Sample}}} \right)/\left. {\Delta {\text{Abs 1}}00\% {\mathrm{activity}}} \right) \\ & \quad \times {1}00 \\ \end{aligned} $$

where ∆Abs indicates the difference between absorbance values after 10 and initial time.

### Real time-PCR

McA-RH7777 and HepG2 cells were seeded in 35 mm culture dishes at a density of 300.000 cells/well. After 24-h of treatments samples were extracted according to the TRIzol isolation reagent protocol (Invitrogen, USA), and the quality and quantity of RNA were evaluated by measuring the absorbance at 260 and 280 nm on a NanoDrop Spectrophotometers (Thermo Scientific). The cDNA was synthesized from 1 µg of total RNA, using High-Capacity cDNA Reverse Transcription Kit (Applied Biosystems, Foster City, CA, USA). mRNA expression were quantified by real time-PCR using SYBR® Green dye (SYBR® Green PCR Master Mix, Applied Biosystems, Foster City, CA, USA) with gene‐specific primers (Supplemental Table 3). Data were compared between samples according to a comparative threshold cycle (2 ^− ΔΔct^) method and normalized to *β‐ACTIN*.

### Statistical analysis

Data are reported as mean ± standard deviation (SD) and percentage (%). Based on a previous study conducted on a Calabrian population with hepatic steatosis (CAP score 285 ± 40 dB/m) [12], to detect a CAP score reduction of at least 8% after 12 weeks of treatment, with an effect size (ES = mean CAP difference/baseline SD) of 0.57, with 80% power on a two-sided level of significance of 0.05, a minimum of 44 subjects for each group were required. Considering a 10% of drop-out, we enrolled 98 patients. The statistical power calculation was strictly computed for a two-arm parallel design (OsteoCol® group vs. control group) to evaluate the main effect of the functional sauce on the primary outcome (CAP score change), regardless of the internal compliance-driven subdivisions.

Both intention-to-treat (ITT) and per-protocol (PP) analyses were performed. PP analysis was performed only on participants taking 80% or more of the prescribed treatment. An independent samples t-test and a χ^2^ test were used to compare differences in means and in prevalence, respectively, between the intervention and control groups. Independent unpaired samples student-t test was used also for evaluating the difference between mean changes from baseline between groups. A Pearson’s correlation was performed to identify the variables correlated with the change of CAP score, given that the continuous variables were normally distributed. The General Linear Model (GLM) was employed to adjust the change in CAP score and the improvement in liver steatosis grade for potential confounding variables identified through in the univariate analysis with a *P* < 0.1.

To further describe the response to the functional tomato sauce, several post-hoc analyses were performed in the following participant subgroups: ITT; PP; men; women; normal weight/overweight, obese; age ≥ 60 years; age < 60 years; and presence or absence of with or without metabolic syndrome. For these post-hoc analyses, we performed an independent unpaired samples t-test to compare the percentage change in CAP score between groups. Furthermore, we used a χ^2^ test to compare the improvement in the liver steatosis grade between groups. The missing value from the follow-up visit was replaced with the previously observed value for that subject, i.e., the last observation was carried forward [44]. In the ITT analysis, the combination of observed and imputed data was then analyzed as if there were no missing data. A *p*-value was significant if < 0.05 (two-tailed). All analyses were performed with SPSS 29.0 for Windows (IBM Corporation, New York, NY, United States).

For in vitro study, data are represented as mean ± standard deviation (SD) of at least three independent experiments and analyzed using a two-tailed Student’s t-test and linear regression. *p*-values less than 0.05 were considered significant. Statistical analysis was performed with GraphPad Prism 10.0.

## Results

### Analysis of carotenoid and lycopene content in sauces

Carotenoid levels in Osteocol® sauce were analyzed using HPLC and compared to those in a control sauce (Supplemental Fig. 1).

The control sauce extracts contained 162.4 ± 10 ppm of lycopene and 8.2 ± 4.6 ppm of β-carotene. Additionally, it showed unquantifiable traces of the *all-trans* lycopene isoform. In contrast, Osteocol® extracts had 247.5 ± 3.1 ppm of *all-trans* lycopene and 78.3 ± 4.3 ppm of β-carotene (Supplemental Table 4). In detail, the HPLC data showed that Osteocol® had a total carotenoid content approximately twice that of the control sauce (6.5 mg/g and 3.42 mg/g for the functional and control sauce, respectively). In fact, Osteocol® functional tomato sauce contained 52.4% more lycopene and over 8 times β-carotene than the control sauce. The antioxidant and free radical scavenging activities of the extracted carotenoids were assessed and compared using the DPPH assay. The control sauce showed an antioxidant activity of 13.13 ± 1.25%I, whereas the Osteocol® sauce exhibited a 35% higher inhibition, reaching 17.67 ± 0.52%I (Supplemental Table 4).

The thermal processing of the sauce did not alter the qualitative or quantitative profile of the Osteocol® matrix. Parameters monitored during the digestive procedures and results obtained are showed in Supplemental Fig. 2 and Supplemental Table 2. Specifically, according to UV–Vis spectrophotometric analyses, during the simulated oral phase a carotenoids concentration of ~ 253 ± 46 ppm was already detected as released from 100 g of the digested sauce. Carotenoids bio-accessibility peaked during the gastric phase, reaching a maximum concentration of ~ 286 ± 31 ppm. Subsequently, over 63% of the gastric carotenoid content remained stable and was recovered during the intestinal phase (~ 182 ± 14 ppm) (Supplemental Fig. 2—Supplemetal Table 2). HPLC analyses confirmed this trend and provided superior and more accurate results. Carotenoids in digestive oral phase had concentration of 269.89 ± 36.07 ppm, of which lycopene and β-carotene represented 236.58 ± 32.46 ppm and 33.31 ± 3.61 ppm, respectively. The digestive gastric phase contained 252.81 ± 9.87 ppm of lycopene and 39.19 ± 1.06 ppm of β-carotene, with total carotenoids exceeding 290 ppm concentration. The final intestinal phase still contained ~ 77% of total carotenoids (ppm) with lycopene and β-carotene respectively representing 203.22 ± 25.20 ppm and 22.67 ± 2.66 ppm (Supplemental Fig. 2—Supplemetal Table 2).

### Human study

**Fig. 1:**
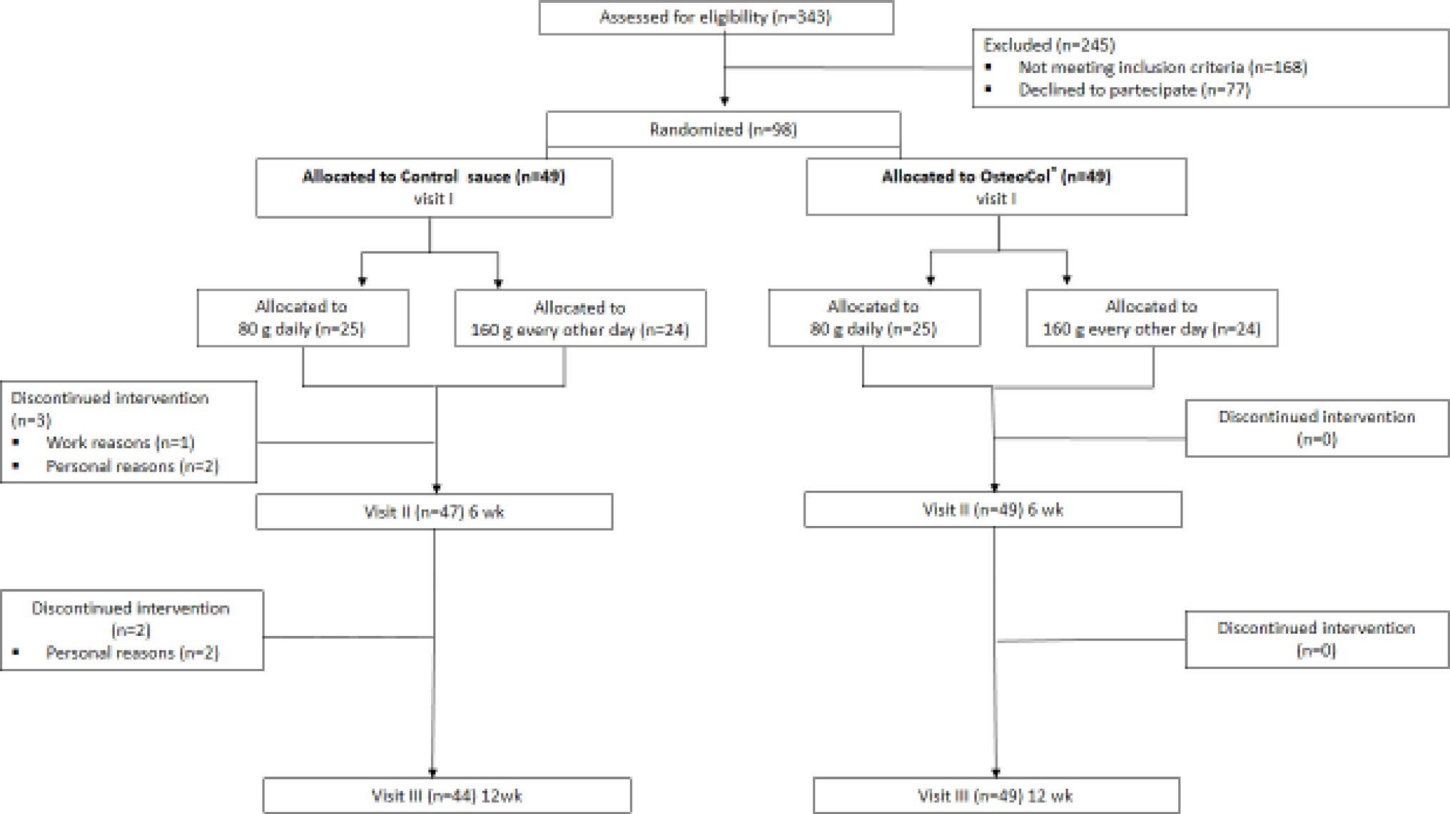
Study flowchart, illustrating the assignment of patients to the two main groups and their subsequent distribution into the respective dosing schedules

Ninety-three participants completed the study. Five subjects discontinued treatment in the placebo group and none of the participants in the functional sauce group (Fig. 1). None of the enrolled patients reported adverse events during the 12 weeks of treatment. The mean age of the population was 55 ± 10 years, and CAP score was 294 ± 36 dB/m. Of the patients enrolled, 54% were male, and 52% of the participants exhibited severe hepatic steatosis (S3 grade).

Table 1 presents the baseline demographic and clinical characteristics of participants with MASLD according to treatment groups. The two groups were comparable for all characteristics except for serum AST levels and LS that were statistically higher in the OsteoCol® group than in the control group (AST: 23 ± 8 vs. 20 ± 5 mg/dL, *p* = 0.02; LSM: 4.7 ± 1 kPa vs. 5.3 ± 1 kPa, *p* = 0.01; respectively).

**Table 1:** Baseline demographic and clinical characteristics of participants according to the treatments

Variables	Control sauce (n = 49)	OsteoCol® (n = 49)	*p*-value
Age (years)	55 ± 10	54 ± 11	0.79
Weight (Kg)	81 ± 13	81 ± 15	0.91
BMI (Kg/m^2^)	29.6 ± 3	29.4 ± 4	0.81
WHR	0.96 ± 0.08	0.96 ± 0.07	0.69
FM (Kg)	25 ± 6	26 ± 8	0.77
CAP score (dB/m)	292 ± 34	295 ± 38	0.73
SD (dB/m)	11 ± 5	12 ± 6	0.56
LSM (kPa)	4.7 ± 1	5.3 ± 1	0.018
IQR/median ratio (%)	16 ± 6	16 ± 5	0.28
Glucose (mg/dL)	91 ± 9	91 ± 11	0.90
Insulin (mU/L)	14 ± 8	15 ± 8	0.54
HOMA-IR	3.2 ± 2	3.4 ± 2	0.59
TC (mg/dL)	191 ± 31	196 ± 38	0.46
TG (mg/dL)	120 ± 42	120 ± 51	0.99
HDL-C (mg/dL)	50 ± 10	52 ± 12	0.49
LDL-C (mg/dL)	116 ± 28	120 ± 33	0.55
Albumin (mg/dL)	4.6 ± 0.2	4.6 ± 0.2	0.89
AST (IU/L)	20 ± 5	23 ± 8	0.028
ALT (IU/L)	27 ± 18	30 ± 20	0.46
γGT (UI/L)	26 ± 20	28 ± 19	0.47
Creatinine (mg/dL)	0.84 ± 0.2	0.84 ± 0.2	0.90
CRP (mg/L)	2.04 ± 2	1.97 ± 2	0.88
*Prevalence*			
Sex (Male, %)	57	51	0.68
MVPA (%)	26	35	0.51
Smokers (%)	33	45	0.30
Obesity (%)	41	43	1
Metabolic syndrome (%)	21	24	0.81
Insulin resistance (%)	57	59	1
Hypertension (%)	37	39	1
Hyperlipidemia (%)	69	57	0.29
Antihypertensive drugs (%)	41	33	0.53
Antiplatelet agents (%)	16	8	0.35
Lipid-lowering agents (%)	31	14	0.08
Liver steatosis S1 grade (%)	33	37	0.74
Liver steatosis S2 grade (%)	14	12	
Liver steatosis S3 grade (%)	53	51	
Liver fibrosis (%)	0	0	/

BMI, body mass index; WHR, waist to hip ratio; FM, fat mass; CAP, controlled attenuation parameter; SD, standard deviation; LSM, liver stiffness measurement; IQR, interquartile range; HOMA-IR, homeostatic model assessment of insulin resistance; TC, total cholesterol; TG, triglycerides; HDL-C, high density lipoprotein cholesterol; LDL-C, low density lipoprotein cholesterol; AST, aspartate aminotransferase; ALT, alanine aminotransferase; γGT, gamma glutamyltransferase; CRP, C-reactive protein. MVPA, moderate-to-vigorous physical activity. Data are represented as mean ± SD or percentage

### Nutrient profile assessment

Supplemental Table 5 shows the dietary intake assessment at baseline, and the changes in dietary intake over the 12-week treatment period for participants based on their allocation. The nutrient profiles of the two groups were comparable at both baseline and the end of the intervention (Supplemental Table 5).

### Clinical characteristics changes at follow-up and outcome of the study

To assess whether different modes of consumption influenced the effect on intrahepatic fat content, the change in CAP score of participants consuming 80 g per day was compared with those consuming 160 g on alternate days. This analysis showed no significant differences in intrahepatic fat reduction between the two OsteoCol® tomato sauce consumption regimens (CAP score: OsteoCol® 80 g per day − 31 ± 51 dB/m vs. OsteoCol® 160 g every other day − 35 ± 36 dB/m; *p* = 0.98). Similarly, no statically significant differences were observed between the two dosing modalities in the control group (CAP score: control sauce 80 g per day − 16 ± 31 dB/m vs. control sauce 160 g every other day − 8 ± 45 dB/m; *p* = 0.92). For this reason, we decided to analyze the data independently of the dosage.

Table 2 shows the changes in anthropometric, instrumental, and laboratory characteristics of the enrolled population after 12 weeks of treatment. At follow-up, the groups were comparable for all the characteristics except for CAP score, and LSM values. In particular, CAP score and LSM reduction were statistically higher in the OsteoCol® group than in the control group (CAP score: − 33 ± 44 dB/m vs. − 12 ± 38 dB/m, *p* = 0.014; LSM: − 0.4 ± 1 kPa vs. 0.2 ± 1 kPa, *p* = 0.008; respectively). Thus, we performed a Pearson’s correlation test to assess the variables correlated to CAP score change. This analysis showed that the CAP score change after 12 weeks of treatment correlated positively with weight change at 12-weeks (r = 0.22; *p* = 0.027) and lipid-lowering treatment (r = 0.28; *p* = 0.004), while it correlated negatively with sex (r = − 0.20; *p* = 0.041) and CAP score levels at baseline (r = − 0.25; *p* = 0.011). No significant correlation was observed with all other parameters analyzed. The GLM test demonstrated that the change in CAP score remained statistically significant even after adjusting for confounding variables between the active treatment and control groups (CAP score: − 35 ± 5 dB/m vs. − 10 ± 5 dB/m, *p* = 0.002; Table 2).

**Table 2:** Changes in clinical characteristics of participants according to the treatments after 12 weeks (intention to treat analysis)

Variables	Control sauce (n = 49)	OsteoCol® (n = 49)	*p*-value
Adherence to treatment (%)	95 ± 10	95 ± 10	0.71
Weight (Kg)	− 2.4 ± 2	− 2.0 ± 2	0.38
BMI (Kg/m^2^)	− 0.89 ± 0.9	− 0.73 ± 0.8	0.34
WHR	− 0.03 ± 0.6	− 0.04 ± 0.4	0.83
FM (Kg)	− 3.7 ± 3	− 4.3 ± 3	0.34
CAP score (dB/m)	− 12 ± 38	− 33 ± 44	0.014
^a^CAP score (dB/m)	− 10 ± 5	− 35 ± 5	0.002
LSM (kPa)	0.2 ± 1	− 0.4 ± 1	0.008
Glucose (mg/dL)	− 1.9 ± 8	0.1 ± 7	0.21
Insulin (mU/L)	− 4.4 ± 6	− 4.7 ± 7	0.86
HOMA-IR	− 1.1 ± 2	− 1.0 ± 2	0.94
TC (mg/dL)	− 8 ± 23	− 12 ± 30	0.52
TG (mg/dL)	5 ± 49	4 ± 46	0.89
HDL-C (mg/dL)	− 2 ± 7	− 3 ± 6	0.55
LDL-C (mg/dL)	− 7 ± 21	− 10 ± 27	0.62
Albumin (mg/dL)	0.006 ± 0.2	− 0.01 ± 0.2	0.67
AST (IU/L)	− 1 ± 5	− 2 ± 7	0.64
ALT (IU/L)	− 5 ± 13	− 6 ± 15	0.88
γGT (UI/L)	− 2 ± 9	− 5 ± 10	0.22
Creatinine (mg/dL)	0.003 ± 0.009	− 0.007 ± 0.008	0.57
CRP (mg/L)	− 0.3 ± 2	− 0.2 ± 3	0.79

^a^Adjusted for sex, lipid-lowering medication, CAP score, LS, serum AST levels at baseline and body weight change at follow-up

BMI, body mass index; WHR, waist to hip ratio; FM, fat mass; CAP, controlled attenuation parameter; LSM, liver stiffness measurement; HOMA-IR, homeostatic model assessment of insulin resistance; TC, total cholesterol; TG, triglycerides; HDL-C, high density lipoprotein cholesterol; AST, aspartate aminotransferase; ALT, alanine aminotransferase; γGT, gamma glutamyl transferase; CRP, C-reactive protein. Data are represented as mean ± SD

**Fig. 2:**
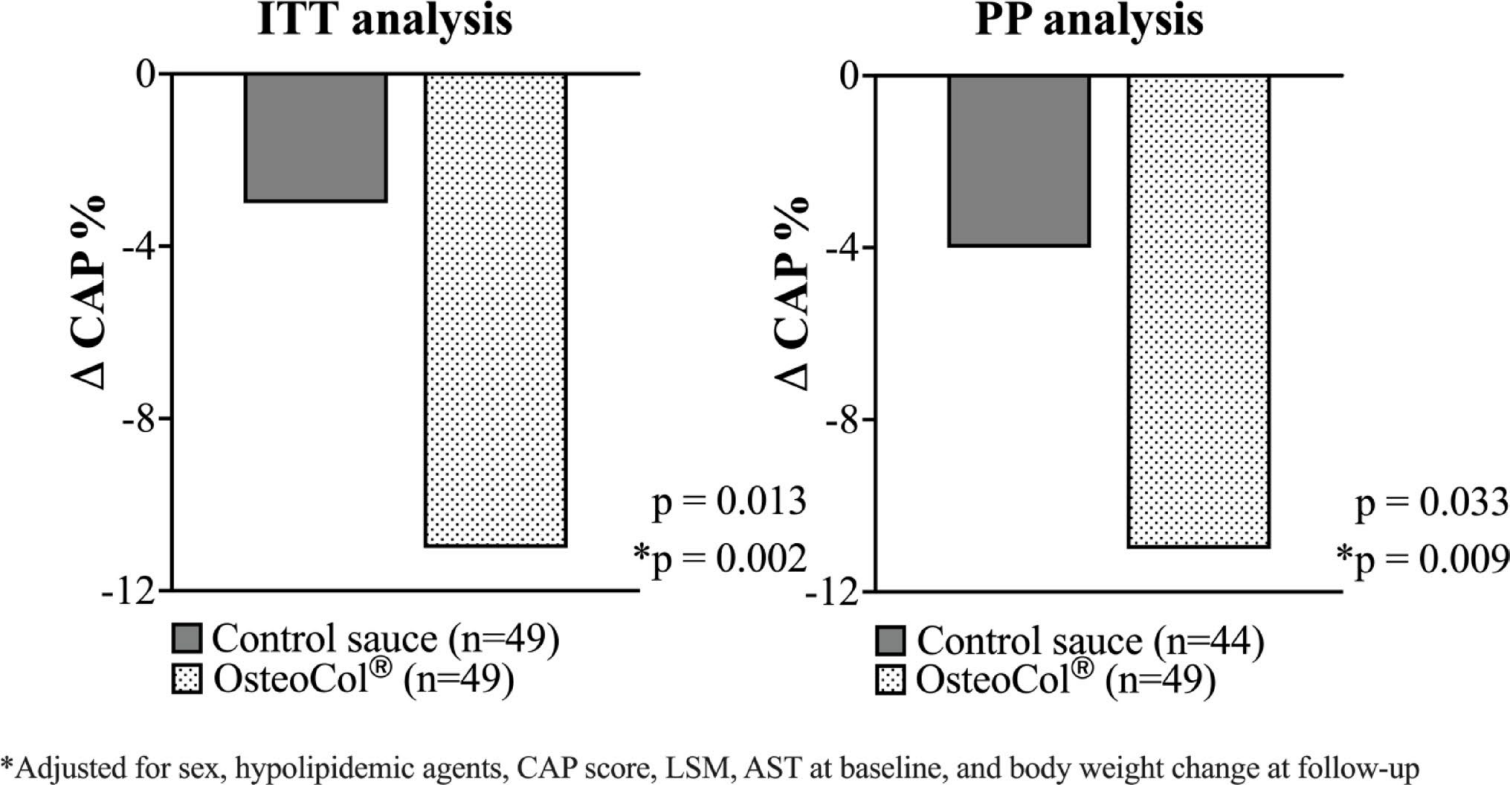
Percentage reduction in CAP score after 12 weeks of treatment in Intention-To-Treat (ITT) as well as *Per Protocol* (PP) analyses

Figure 2 shows the percentage reduction in CAP score across treatments in both ITT and PP analyses. In both analyses, we found that subjects consuming the functional sauce had a significantly greater percentage reduction in CAP score compared to those receiving the placebo (ITT analysis: − 11% vs. − 3%, *p* = 0.013; PP analysis: − 11% vs. − 4%, *p* = 0.033; Fig. 2). These reductions remained statistically significant even after adjustment for confounding variables (Fig. 2).

**Fig. 3:**
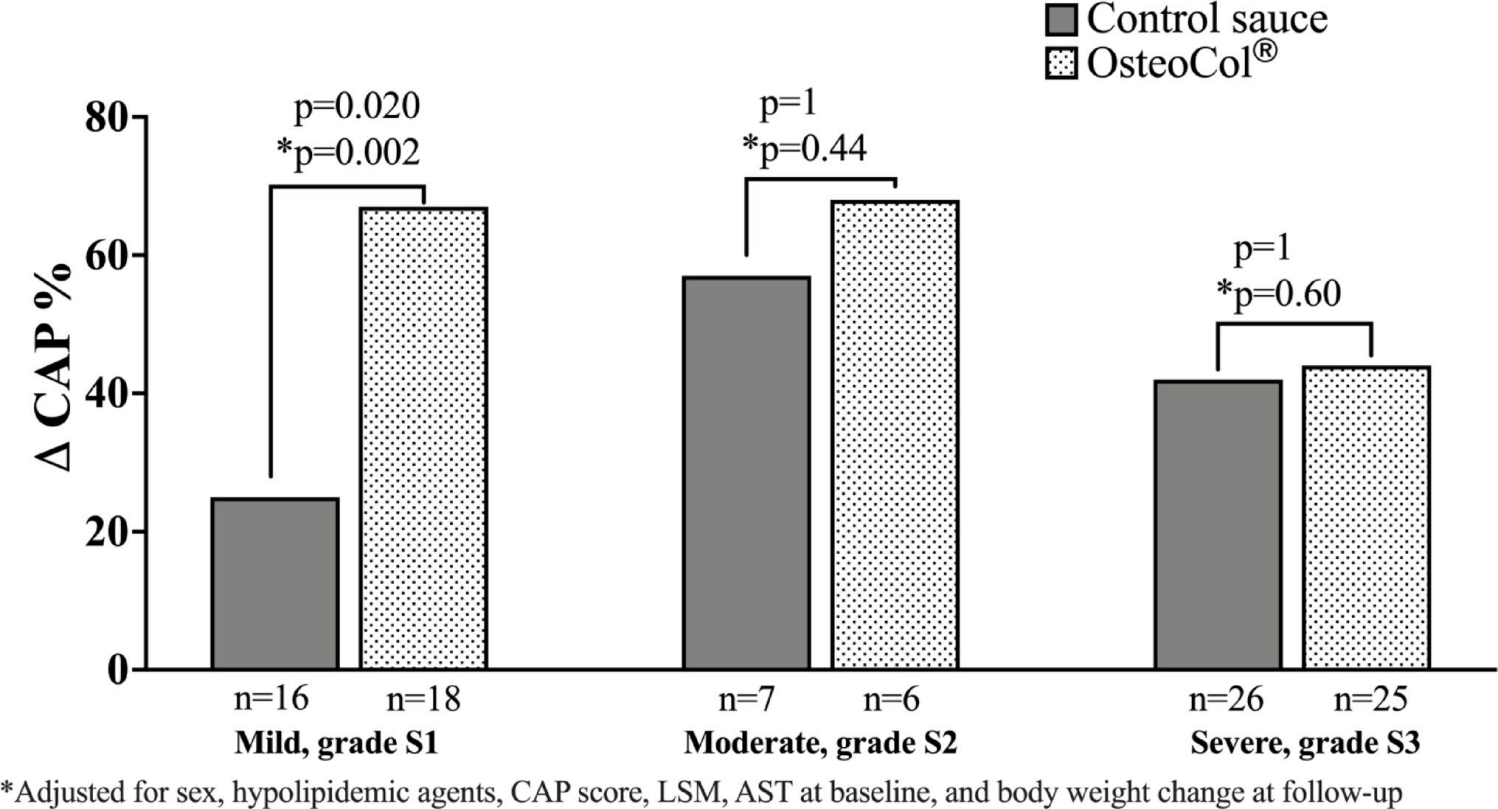
ITT analysis: prevalence of improvement of hepatic steatosis grade according to the interventions and severity of liver steatosis at baseline

The prevalence of improvement of hepatic steatosis grade according to the treatments and severity of liver steatosis at baseline was reported in Fig. 3. In the group of participants with mild hepatic steatosis (S1 grade), we found that individuals consuming functional tomato sauce had a significantly greater improvement in the stage of liver steatosis compared to placebo (67% vs. 25%, *p* = 0.020; respectively). The improvement of liver steatosis grade remained statistically significant even after adjustment for confounding variables (*p* = 0.002; Fig. 3). No statistically significant differences between groups were observed in participants with moderate (S2 grade) and severe hepatic steatosis (S3 grade)(Fig. 3).

**Fig. 4:**
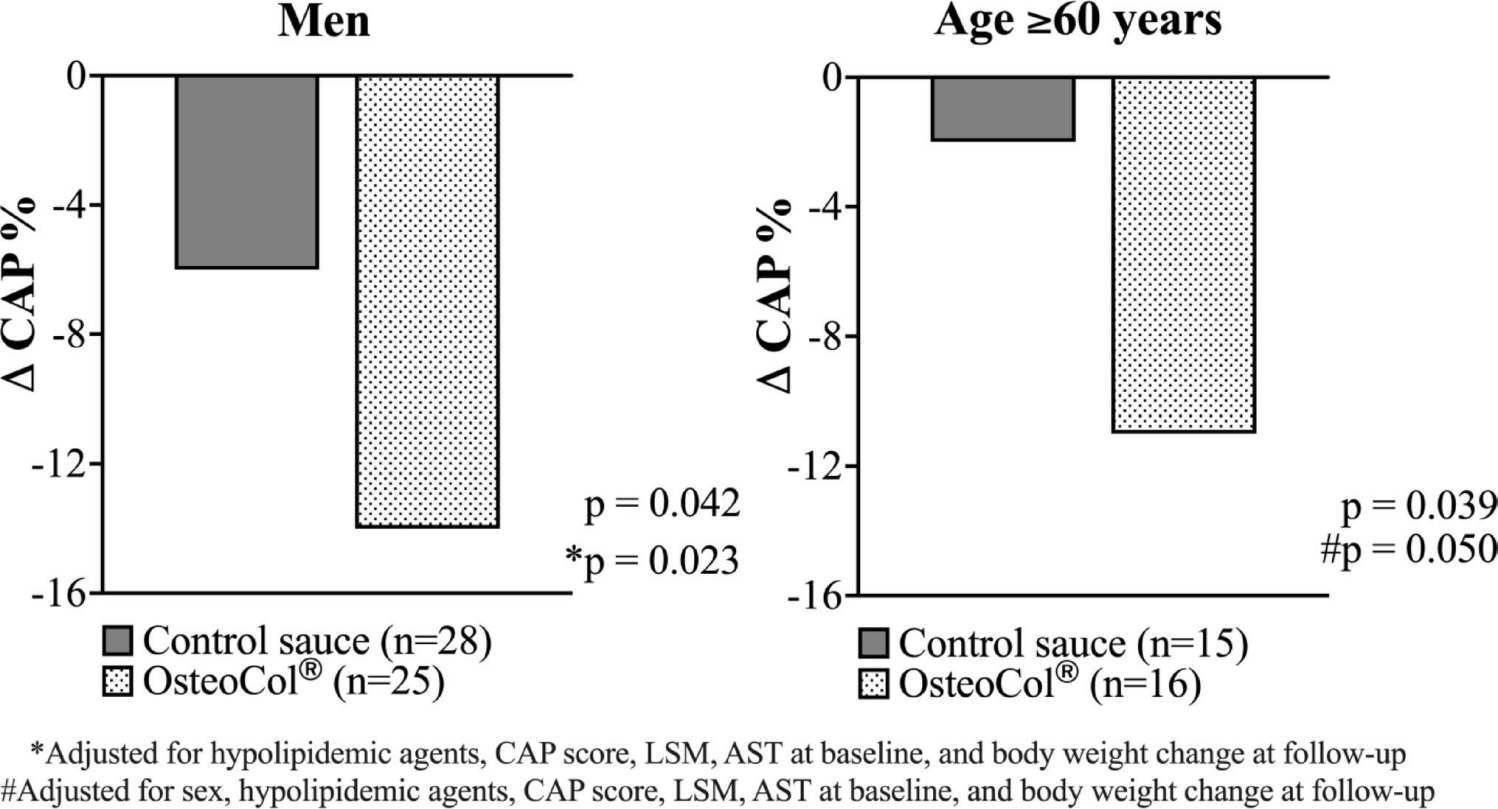
Subgroup analyses: percentage reduction in CAP score after 12 weeks of treatment in men and individuals over 60 years old

**Fig. 5:**
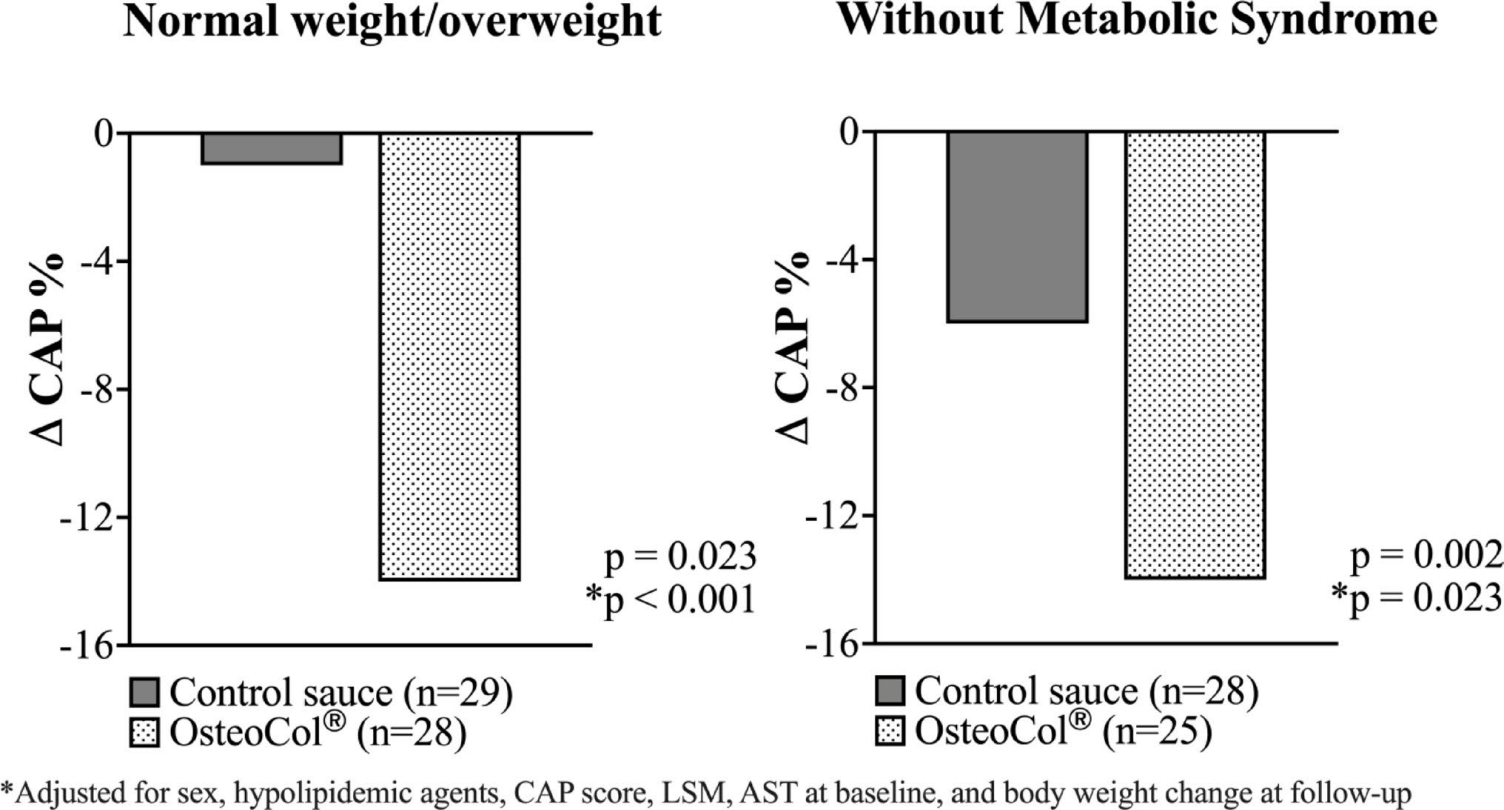
Subgroup analyses: percentage reduction in CAP score after 12 weeks of treatment in overweight/obese individuals and in participants without metabolic syndrome

Intrahepatic fat reduction in the subgroup analyses based on the interventions was reported in Figs. 4 and 5. In particular, the percentage reduction in CAP score was statistically significant in men and in participants older than 60 years, even after adjustment for confounding variables (Fig. 4).

Finally, in the subgroup analysis, we observed a significant reduction of 13% in CAP score in overweight/obese subjects and 11% in subjects without MS who consumed functional tomato sauce, compared to the control group (Fig. 5). The percentage reduction in CAP score remained statistically significant in both subgroup analyses even after adjustment for sex, lipid-lowering medication, CAP score, LSM, serum AST levels at baseline and body weight change at follow-up (Fig. 5).

### In vitro study

#### Characterization of carotenoids micellar suspension for in vitro study

To verify the internalization of lycopene, the major carotenoid present in the extract, into the micellar formulation for in vitro studies, both the free lycopene extract and its encapsulated form were analyzed using a UV–Vis spectrophotometer. Lycopene extracted from functional sauce showed a characteristic profile with three main peaks which, in decreasing order of absorbance, were at ~ 470 nm, ~ 510 nm, and ~ 440 nm, respectively (Supplemental Fig. 3—Panel A blue line). The development of a micellar formulation incorporating lycopene led to the displacement of the conventional carotenoid profile, replacing it with a new distinctive peak, specific to the micellar complex and observed at a wavelength ~ 350 nm (Supplemental Fig. 3—Panel A red line).

Moreover, with the aim to simulate the accelerated physiological photodegradation processes of lycopene, the aforementioned lycopene samples were both exposed to UV radiation (Supplemental Fig. 3—panel B and panel C). The acquisition of absorbance values showed that approximately 50% of the free lycopene was degraded after 5 min exposure to UV radiation, reaching a photodegradation of 77% after 10 min exposure. On the contrary, the micellar lycopene formulation detectable at 344 nm underwent a photodegradative alteration of 2% in 10 min of exposure to UV rays. In the same formulation, even the not totally encapsulated portion of lycopene, quantified at 478 nm, evidenced a partial photoprotective effect, keeping approximately 77% of the molecule unchanged after 10 min (Supplemental Fig. 3—panel B and panel C).

#### Osteocol® micellar suspension reduces the accumulation of neutral lipids in hepatocytes

To assess the effect of the Osteocol® extract formulated as a carotenoids micellar suspension (CMS) on cell viability, MTT assays were performed on McA-Rh7777 and HepG2 hepatic cell lines. The results showed no evidence of cytotoxicity under the tested conditions (Supplemental Fig. 4).

Since empty micelles (Tween 80®) represented the vehicle control for the OsteoCol® CMS, we first evaluated whether they could affect intracellular neutral lipid accumulation. Oil Red O staining showed that empty micelles increased neutral lipid accumulation compared with untreated cells (Supplementary Fig. 5). Therefore, all subsequent analyses were performed by comparing OsteoCol® CMS-treated cells with empty micelle-treated cells, in order to specifically assess the effect of the carotenoid-containing micellar suspension.

**Fig. 6:**
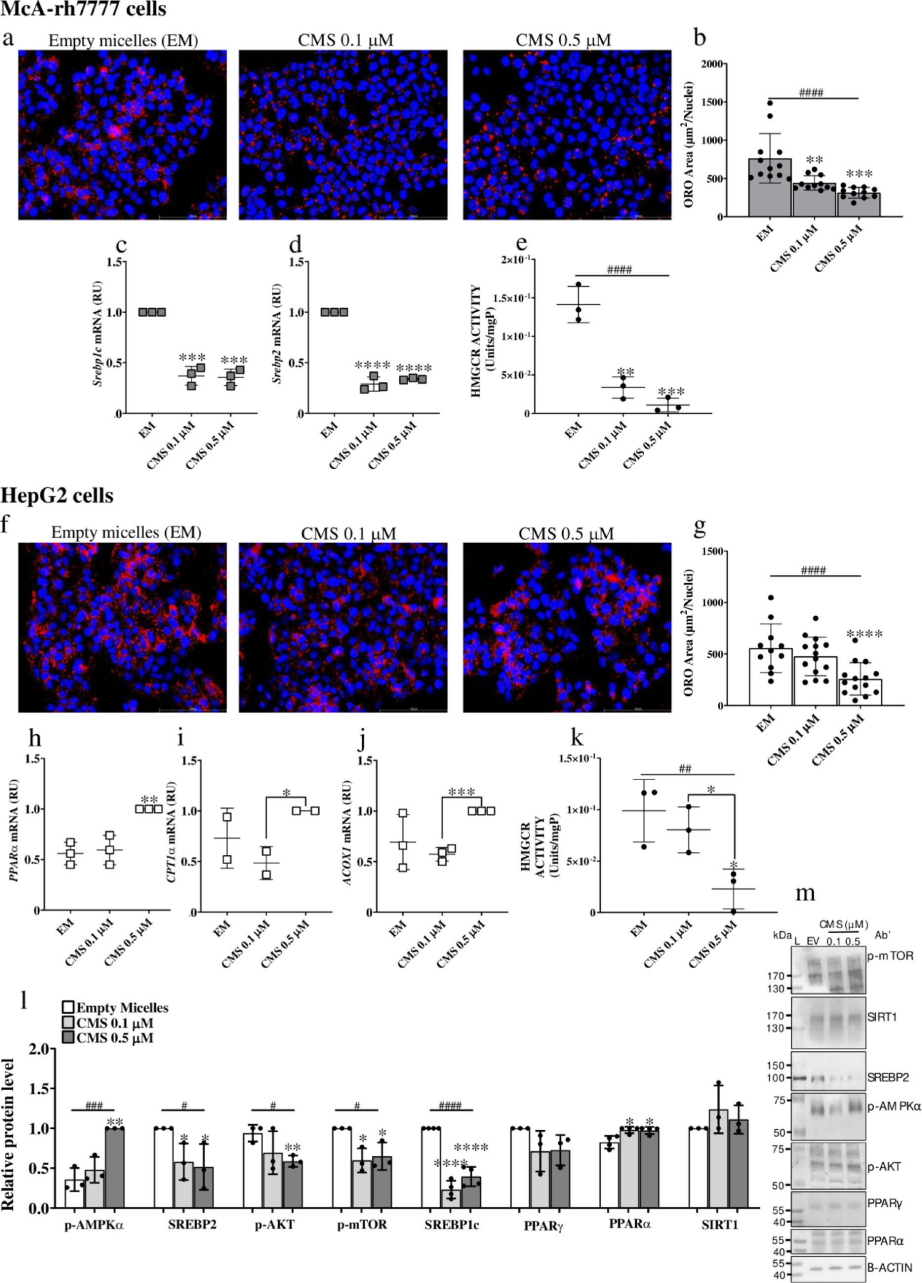
CMS lowers neutral lipids content and reduces cholesterol and triglyceride markers on hepatocytes cells. **A**, **F** Intracellular lipid content was measured by Oil red-O staining and **B**, **G** ORO area quantified by Image J. **C**, **D**, **H**, **I**, **J** mRNA expression levels of *Srebp-1c*, *Srebp-2 PPARa, CPT1a* and *ACOX1* were measured using real-time PCR. Data were analyzed using the 2^-ΔΔCt method and normalized to *β-ACTIN*. **E** HMGCR activity was analyzed using HMG-CR Inhibitory Activity Assay; **I**, **J** Protein abundance in total cell lysates was assessed by western blotting and relative quantification of p-AMPK, SREBP2, p-AKT, p-mTOR, SREBP-1c, PPARγ, PPARα and SIRT1 expression. Data are represented as mean ± SD of three independent experiments and *p*-values are calculated by Student’s t-test (*p < 0.05; **p < 0.01; ***p < 0.001; ****p < 0.0001) and Linear regression (^#^*p* < 0.05; ^##^*p* < 0.01; ^###^*p* < 0.001; ^####^*p* < 0.0001). *Abbreviations*: RU = Relative Unit

Initially, we examined the effect of CMS on neutral lipids by incubating the rat hepatic cell line McA-Rh7777 with different concentrations of the micellar suspension (0.1 and 0.5 µM) for 24 h, using Oil Red O staining. Data showed a significant reduction in neutral lipid content, as triglycerides and cholesterol esters (Fig. 6A, B), compared to cells treated with empty micelles. Next, we assessed the mRNA levels of the *SREBP-1c* gene, involved in triglyceride synthesis, observing a dose-dependent decrease (*p* < 0.001) (Fig. 6C). We extended the investigation to cholesterol metabolism, measuring the activity of the enzyme HMG-CoA Reductase (HMGCR) and the expression of the transcription factor *SREBP*-2 in the same cell line. We demonstrated a dose-dependent reduction in both *SREBP-2* expression and HMGCR activity compared to control (*p* < 0.0001 and *p* < 0.0001, respectively; Fig. 6C, D). These results indicate a modulation of the regulation of triglyceride and cholesterol synthesis in McA-Rh7777 cells. Subsequently, to confirm the results in a physiological cellular system, we tested the effects of CMS on immortalized human hepatocytes HepG2. The results confirmed the reduction in neutral lipids after incubation with the CMS compared to the control (empty micelles) (Fig. 6E, F). To further investigate the specific contribution of lycopene to this effect, HepG2 cells were treated with isolated all-trans lycopene under the same experimental conditions used for OsteoCol® CMS. Oil Red O staining showed that isolated all-trans lycopene reduced intracellular neutral lipid accumulation in HepG2 cells, suggesting that lycopene may contribute, at least in part, to the lipid-lowering effect observed with the OsteoCol® carotenoid micellar suspension (Supplemental Fig. 6).

To determine whether the CMS alters the expression of proteins related to cholesterol synthesis, we assessed the enzymatic activity of HMGCR (Fig. 6G) and total cellular protein abundance of AMPK and SREBP-2 in total cell lysates (Fig. 6I). HMGCR activity showed a dose-dependent reduction (*p* = 0.0089) compared to control, and the higher dose of the CMS (0.5 µM) presented lower activity than the lower dose (*p* < 0.05; Fig. 6G). Total cellular AMPK protein abundance increased, whereas total cellular SREBP-2 protein abundance decreased after CMS treatment (*p* = 0.0017 and *p* = 0.0363, respectively; Fig. 6I, J). Furthermore, to assess the effects of the CMS on triglyceride metabolism in the HepG2 cell line, we examined the protein expression levels of pathways involved in de novo lipogenesis, specifically phosphorylated-AKT (p-AKT) and the mechanistic target of rapamycin (p-mTOR). The results showed a statistically significant reduction in a dose-dependent manner compared to the control (*p* = 0.04 and *p* = 0.017 respectively; Fig. 6I, J). In addition, total cellular SREBP-1c protein abundance was reduced in a dose-dependent manner, suggesting a modulation of lipogenesis-related pathways (*p* < 0.0001; Fig. 6I, J).

We also measured gene and protein levels of PPARα, observing an increase after incubation with the CMS (*p* = 0.002 and *p* < 0.05, respectively; Fig. 6H, J). Conversely, SIRT1 and PPARγ levels were not modulated by the treatment with the micellar suspension containing lycopene (Fig. 6I, J). These results suggest that the CMS can influence cholesterol and triglyceride metabolism, promoting an increase in β-oxidation.

## Discussions

Our study investigated the effects of a functional tomato sauce, naturally enriched in carotenoids, on hepatic fat accumulation in adults with MASLD. Tomatoes and their derivatives have always been a fundamental pillar of the Mediterranean Diet and, in recent years, have become the object of growing interest by the scientific community due to the benefits attributed to the bioactive compounds they contain, in particular carotenoids. It is known that the concentration of these substances in tomatoes can be influenced by multiple external factors, such as industrial transformation processes, storage conditions and consumption methods [45]. The data emerging from this study, in line with the evidence already present in the literature, show that OsteoCol® sauce contains a significantly higher amount of lycopene than the control sauce. In detail, the extracts obtained from OsteoCol® have approximately 52% more lycopene and concentrations of β-carotene 8 times higher than the control sauce. Furthermore, OsteoCol® tomato sauce, obtained from tomatoes ripened on the vine, has a lycopene content of ~ 5 mg per gram of product, an extraordinarily high concentration that exceeds by more than 30 times the values commonly reported in the literature for tomato sauces and concentrates, which rarely exceed 15 mg/100 g [46, 47].

In this context, it is important to distinguish between total lycopene and its isomeric forms. OsteoCol® contained exclusively all-trans lycopene, whereas the control sauce exhibited several lycopene isoforms. This aspect may be biologically relevant, as all-trans lycopene is considered a marker of freshness in tomato-based products and is characterized by greater chemical stability and a linear molecular configuration, which may favor its ordered incorporation into lipid membranes and contribute to antioxidant and membrane-protective effects [48].

Although cis-isomers are generally associated with higher bioavailability [48, 49], the predominance of all-trans lycopene in OsteoCol® may help explain its greater antioxidant activity. Furthermore, our in vitro experiments performed with isolated all-trans lycopene demonstrated a reduction in intracellular neutral lipid accumulation in HepG2 cells, suggesting that all-trans lycopene may also exert direct biological effects independently of its possible conversion into cis-isomers, and may therefore contribute, at least in part, to the lipid-lowering effect observed with OsteoCol®.

However, since OsteoCol® represents a complex carotenoid-rich matrix, potential contributions from other components of the food matrix cannot be excluded.

Moreover, in this RCT, we demonstrated for the first time that regular consumption of OsteoCol® tomato sauce is effective in reducing intrahepatic fat accumulation in adult subjects with MASLD, thus leading to disease regression. In particular, after 12 weeks of treatment, participants in the intervention group showed a significant 11% reduction in the amount of intrahepatic fat compared to the 3% observed in the control group. This finding is in line with that reported in other studies with nutraceuticals [12], and can be considered clinically relevant. It is important to underline that, although liver stiffness values were within the normal range, they were also significantly reduced in the treatment group compared to the control group, suggesting beneficial effects of this functional sauce on the entire liver parenchyma.

In fact, it has been described that the intake of carotenoids can significantly reduce the risk of suffering from hepatic diseases [50]. In particular, it has been demonstrated that lycopene at the liver level is able to prevent fibrosis by down-regulating some fibrosis markers such as transforming growth factor β 1 and α-smooth muscle actin, and by reducing oxidative stress and inflammation [51]. These results are very interesting as they provide tangible evidence of how nutritional strategies that also include the use of functional foods can be a valid aid in reducing fat accumulation in the liver and preventing related complications.

Our results confirm the most robust epidemiological evidence on the relationship between dietary carotenoids and MASLD. One study demonstrated that serum concentrations of total carotenoids and lycopene were associated with improvement in hepatic steatosis over 3 years [31]. Furthermore, lycopene consumption has been shown to play a protective role against liver diseases, including alcoholic fatty liver disease and other chronic liver diseases [33]. Furthermore, studies on animal models of MASLD have demonstrated the beneficial effects of lycopene supplementation in improving the state of inflammation and hepatic steatosis [19, 27, 52–54].

Although the results obtained in our study on both cellular and human models confirm the effects highlighted in animal models, we do not find a reduction in inflammation parameters. These data are not surprising, as the patients included in the study presented low baseline CRP levels, indicating a mild clinical condition without signs of disease progression. This was further supported by the absence of liver fibrosis observed in our study population.

Subgroup analyses revealed that the effect of OsteoCol® consumption on CAP score reduction was more pronounced in men, patients over 60 years of age, those with mild MASLD, normal weight and overweight, and without MS (Figs. 3, 4, and 5). The effect of functional tomato sauce consumption in men is plausible, since sex differences in metabolic, inflammatory, oxidative and hormonal pathways, as well as in the expression of genes related to hepatic metabolism and triglyceride accumulation are well known [55], which may explain the sex disparity in MASLD prevalence and different responses to treatment [56]. As widely recognized, oxidative stress plays a crucial role in the pathogenesis of fatty liver disease [57], and at the same time the aging process is also associated with an increase in oxidative stress [51]. It is also widely documented that lycopene is a potent antioxidant [58]. Therefore, the increase in oxidative stress that occurs in both fatty liver disease and elderly individuals could be counteracted by carotenoids in functional tomato sauce. This mechanism could help explain the greater efficacy observed in reducing liver fat accumulation in elderly subjects following the intake of functional tomato sauce. Moreover, the subgroup analysis indicates that individuals with lower intrahepatic fat content (S1 grade) had greater benefits from OsteoCol® treatment. This finding is plausible, as weight loss, dietary interventions, and physical activity are known to reverse hepatic steatosis [59], and this effect tends to be more evident in individuals with less intrahepatic fat accumulation [60]. Furthermore, in our RCT, we observed that normal weight and overweight individuals and those without MS showed a significant reduction in CAP score following OsteoCol® treatment. This finding is plausible given the less pronounced metabolic alterations in these individuals, which could allow a more effective response to nutritional interventions [60], such as carotenoid supplementation.

In the present RCT, no effect on blood cholesterol levels was observed, in contrast to the findings reported by Ferro et al*.* in a previous RCT conducted using OsteoCol® tomato sauce [34]. This discrepancy could be attributable both to differences in the dosage of tomato sauce consumed (80 g/day *vs.* 150 g/day), and to the characteristics of the population enrolled (subjects with MASLD *vs.* subjects with polygenic hypercholesterolemia) [34]. Furthermore, at the end of the study we did not find a statistically significant reduction in body weight, nor changes in eating habits between the two groups, suggesting that other mechanisms independent of body weight reduction and changes in eating habits may have contributed to the reduction of intrahepatic fat.

To understand the molecular mechanisms underlying the reduction of the CAP score in humans, we tested the effect of carotenoid extracted from the OsteoCol® sauce conveyed in a micellar formulation that uses a surfactant [16] to preserve lycopene also from photodegradation on murine and human liver cell lines. The findings obtained from the in vitro study support the hypothesis that this carotenoid-rich matrix may directly modulate lipid metabolism in hepatocytes. In particular, OsteoCol® CMS reduced intracellular lipid accumulation and modulated pathways involved in lipogenesis, cholesterol synthesis, and fatty acid oxidation. In parallel, experiments performed with isolated all-trans lycopene showed a reduction in intracellular neutral lipid accumulation in HepG2 cells, suggesting that lycopene may contribute, at least in part, to the lipid-lowering effect of OsteoCol®. However, since OsteoCol® is a complex carotenoid-rich food matrix, the contribution of β-carotene, other carotenoids, and additional tomato-derived bioactive compounds cannot be excluded.

In particular, OsteoCol® micellar extract reduced the expression of SREBP-1c, a key transcription factor in the regulation of lipogenesis, and simultaneously decreased AKT/mTOR signaling pathways, known to drive hepatic lipid synthesis. The extract decreased SREBP-1c levels, suggesting a reduction in de novo lipogenesis, and reduced both HMG-CoA reductase activity and SREBP-2 levels, indicating a potential effect on cholesterol synthesis. Moreover, OsteoCol® treatment was associated with increased p-AMPK and reduced p-AKT and p-mTOR levels, together with increased PPARα expression and modulation of its metabolic targets CPT1a and ACOX1. Overall, these findings suggest that OsteoCol® may contribute to the reduction of hepatic lipid accumulation through the coordinated modulation of lipogenesis, cholesterol synthesis, and fatty acid oxidation-related pathways.

Furthermore, while our findings focus primarily on the direct protective mechanisms of lycopene within hepatocytes, the potential systemic involvement of the gut–liver axis warrants consideration. Emerging evidence indicates that dietary carotenoids, including lycopene, can positively modulate the composition of the intestinal microbiota and strengthen the gut mucosal barrier function [61, 62]. By promoting a favourable microbial balance and preserving tight junction integrity, lycopene administration may mitigate intestinal inflammation [61, 62], thereby indirectly attenuating the development and progression of MASLD.

This RCT has both limitations and strengths. Among the limitations, although the sample size was adequate to detect the expected effect on CAP score, the study was conducted at a single center, which may limit the generalizability of the results. Furthermore, the 12-week duration may not be sufficient to evaluate the long-term effects of this nutritional intervention on hepatic metabolism and progression of hepatic steatosis in subjects with MASLD. Larger, longer-term studies will be needed to confirm the beneficial effects of OsteoCol® observed. Furthermore, the study population consisted exclusively of subjects with MASLD and, therefore, the applicability of the results to patients with more advanced stages of liver disease (such as fibrosis or MASH) remains to be established. Another limitation is the absence of oxidative stress and inflammatory biomarkers, as well as the lack of direct assessment of plasma carotenoid absorption, which would have allowed a more direct correlation between OsteoCol® intake and hepatic effects. Although previous studies demonstrated the bioavailability of Osteocol® carotenoids [34], the inclusion of circulating lycopene and other carotenoid measurements in future trials would further strengthen the mechanistic interpretation of the observed hepatic benefits.

Moreover, although all participants adhered to recommendations based on the Mediterranean diet, the potential influence of uncontrolled dietary or environmental factors on liver fat content cannot be completely ruled out. Finally, a limitation of the in vitro analysis is that p-AMPK, p-AKT, and p-mTOR were normalized to β-actin as a loading control rather than to their corresponding total proteins. Therefore, these data reflect changes in phosphorylated protein abundance and not phosphorylated/total protein ratios. Furthermore, the independent effects of β-carotene alone were not investigated in this cellular model.

Despite these limitations, the study has several strengths. First, none of the participants reported adverse events during the 12-week intervention, confirming the safety and tolerability of OsteoCol® tomato sauce in MASLD patients. Another aspect of great relevance was the high compliance to nutritional treatment, suggesting that the intake of this functional tomato sauce may represent a truly effective strategy to reduce hepatic fat accumulation in this population. Furthermore, the beneficial effects of OsteoCol® tomato sauce on intrahepatic fat reduction appear to be independent of the specific dosing regimen. This flexibility in the intake may improve the practical applicability of the intervention, since patients can choose the most convenient mode of consumption without compromising its efficacy. Another strength of the study is the experimental design, which minimizes the risk of bias and increases the robustness of our results. Furthermore, hepatic steatosis was assessed using VCTE, which provides an objective, non-invasive, and highly reproducible measurement of intrahepatic fat content [63, 64], further increasing the quality and reliability of the results obtained.

Another strength of this RCT was the use of dietary recommendations based on the Mediterranean diet, without caloric restrictions, for both groups, which helped to minimize the potential impact of confounding dietary variations between groups. Finally, the findings observed in humans were supported by in vitro studies conducted on two different hepatic cell lines, which explored the potential molecular mechanisms underlying the observed effect, providing further evidence of a causal link between OsteoCol® consumption and the reduction of intrahepatic fat content.

In conclusion, this study demonstrates that nutritional approaches involving functional foods rich in bioactive compounds, such as tomato sauce naturally enriched in carotenoids, may represent an effective strategy to reduce intrahepatic fat content in adults with MASLD. These results open new perspectives for the application of personalized nutritional interventions in the prevention and management of hepatic steatosis in patients with MASLD.

## Supplementary Information

Below is the link to the electronic supplementary material.

Supplementary Material 1

## Data Availability

The datasets used and/or analysed during the current study are available from the corresponding author on reasonable request.
