# Supplementary material for: A carotenoid-rich functional tomato sauce (OsteoCol®) reduces liver fat content in adults with metabolic dysfunction-associated steatotic liver disease: a randomized clinical trial
Source: Eur J Nutr. 2026 Jul 3;65(5):189. doi: 10.1007/s00394-026-04049-w (PMC13331848; doi:10.1007/s00394-026-04049-w)
Supplement: Supplementary file 1 — Supplementary Material 1 [file 394_2026_4049_MOESM1_ESM.docx]

**Supplementary tables and Figures**

| **Supplemental Table 1**. Nutritional profiles of the functional and control tomato sauces. | | | | | | |  |
| --- | --- | --- | --- | --- | --- | --- | --- |
| Nutrients | | Osteocol^®^ (100g) | | | Control Sauce (100g) | |  |
| Energy (kcal) | | 37 | | | 32 | |  |
| Total Carbohydrates (g) | | 7.2 | | | 5.6 | |  |
| Sugars (g) | | 7 | | | 4.3 | |  |
| Fiber (g) | | 1 | | | 1.6 | |  |
| Proteins (g) | | 1.1 | | | 1 | |  |
| Total Fats (g) | | 0.4 | | | 0.2 | |  |
| SFA (g) | | 0 | | | 0 | |  |
| Salt (g) | | 0 | | | 0 | |  |
| SFA, saturated fatty acids | | | | | | |  |
| **Supplemental Table 2.** Analytical quantification of carotenoids in all in vitro digestive phase of the functional tomato sauce. | | | | | | | |
| **Sample** | **UV-Vis Spectrophotometer** | | **HPLC** | | | | |
|  | ppm | | Carotenoids (ppm) | Lycopene (ppm) | | β-carotene (ppm) | |
| Oral Phase | 253±46 | | 269.89±36.07 | 236.58±32.46 | | 33.31±3.61 | |
| Gastric Phase | 286±31 | | 292.00±10.93 | 252.81±9.87 | | 39.19±1.06 | |
| Intestinal Phase | 182±14 | | 225.89±22.54 | 203.22±25.20 | | 22.67±2.66 | |

| **Supplemental Table 3.** Primers for qRT-PCR analysis | | |
| --- | --- | --- |
| **Gene symbol** | **Forward (5′–3′)** | **Reverse (3′–5′)** |
| *Rat Srebp-1c* | CCAGCCTTTGAGGATAACCA | TGCAGGTCAGACACAGGAAG |
| *Rat Srebp-2* | CAGGGGTCTTCAGCATGATT | GGCAAGAGACCTGAGTCCTG |
| *Rat Actb* | GCCCTGAGGCACTCTTCCA | TTGCGGATGTCCACGTCA |
| *Human HMGCR* | AGTTATCCTGCTCCCCACCTCCGG | GAAGGTCCAACGGCAGGACACCT |
| *Human SREBP-1C* | TCCAGCTACTACTCGTGTGAC | CCCTCTGTTGGGAATTGTTCTG |
| *Human SREBP-2* | AGGCAGGCTTTGAAGACGAA | GTACATCGGAACAGGCGGAT |
| *Human PPARa* | CACTTAAAATGCCACCAGCA | AGAGAAGCCGATGGAAATGA |
| *Human CPT1a* | TTGGAGTTCTTCGTAGCTTGTG | CAGGACAGACATATTGGTTGTG |
| *Human ACOX1* | AAGGGCCATGGACTATTTCC | GACTCCACCCGATGACAGTT |
| *Human ACTB* | TCCCTGGAGAAGAGCTACGA | AGGAAGGAAGGCTGGAAGAG |
| ***Abbreviation****: HMGCR: 3-HYDROXY-3-METYLGLUTARYL-CoA REDUCTASE; SREBP-1C: STEROL REGULATORY ELEMENT BINDING PROTEIN 1c; SREBP-2: STEROL REGULATORY ELEMENT BINDING PROTEIN 2; PPARα: PEROXISOME PROLIFERATOR-ACTIVATED RECEPTOR-ALPHA; CPT1a: CARNITINE PALMITOYLTRANSFERASE 1A; ACOX1: ACIL-CoA OXIDASE; ACTB: ACTIN BETA.* | | |

| Supplemental Table 4*.* Carotenoids, lycopene and β-carotene content of the functional and control tomato sauces. | | | | | |
| --- | --- | --- | --- | --- | --- |
| Sample | Lycopene  (µg/g sauce) | All-trans Lycopene (µg/g sauce) | β-carotene  (µg/g sauce) | Total carotenoids (µg/g sauce) | Antioxidant Activity (I%) |
| Control sauce | 324.77 ± 20 | NA | 16.33 ± 9.3 | ~341 µg | 13.13±1.25 |
| Osteocol^®^ | NA | 494.94 ± 6.14 | 156.55 ± 8.58 | ~651 µg | 17.67±0.52 |
| NA, not applicable. Data are represented as mean ± SD. | | | | |  |

| **Supplemental Table 5**. Dietary intake assessment, and nutrients intake changes during the study (Intention To Treat analysis) | | | | | | | |
| --- | --- | --- | --- | --- | --- | --- | --- |
|  | ***Baseline*** | | | ***Dietary Changes*** | | | |
| **Variables** | **Control sauce (n=49)** | **OsteoCol^®^ (n=49)** | ***p-value*** | **Control sauce (n=49)** | **OsteoCol^®^ (n=49)** | | ***p-value*** |
| Calories Intake (Kcal) | 2465±666 | 2382±485 | 0.49 | -521±560 | | -435±572 | 0.45 |
| Carbohydrates (g) | 267±76 | 266±68 | 0.90 | -50±67 | | -43±75 | 0.63 |
| Animal protein (g) | 55±21 | 51±17 | 0.36 | -10±20 | | -8±23 | 0.57 |
| Vegetable protein (g) | 35±11 | 32±8 | 0.14 | -9±12 | | -6±8 | 0.10 |
| Animal fat (g) | 39±21 | 36±17 | 0.36 | -14±17 | | -11±17 | 0.45 |
| Vegetable fat (g) | 74±28 | 74±22 | 0.87 | -15±35 | | -15±34 | 0.96 |
| SFA (g) | 32±12 | 30±10 | 0.55 | -8±10 | | -7±12 | 0.60 |
| MUFA (g) | 62±23 | 61±16 | 0.78 | -11±26 | | -12±24 | 0.89 |
| PUFA (g) | 21±35 | 15±5 | 0.25 | -10±36 | | -3±7 | 0.19 |
| Cholesterol (g) | 264±102 | 257±10 | 0.73 | -58±98 | | -41±120 | 0.43 |
| Alcohol (g) | 8±9 | 7±10 | 0.46 | -4±8 | | -2±6 | 0.14 |
| SFA, saturated fatty acids; MUFA, monounsaturated fatty acids; PUFA, polyunsaturated fatty acids. Data are represented as mean ± SD or percentage. | | | | | | | |


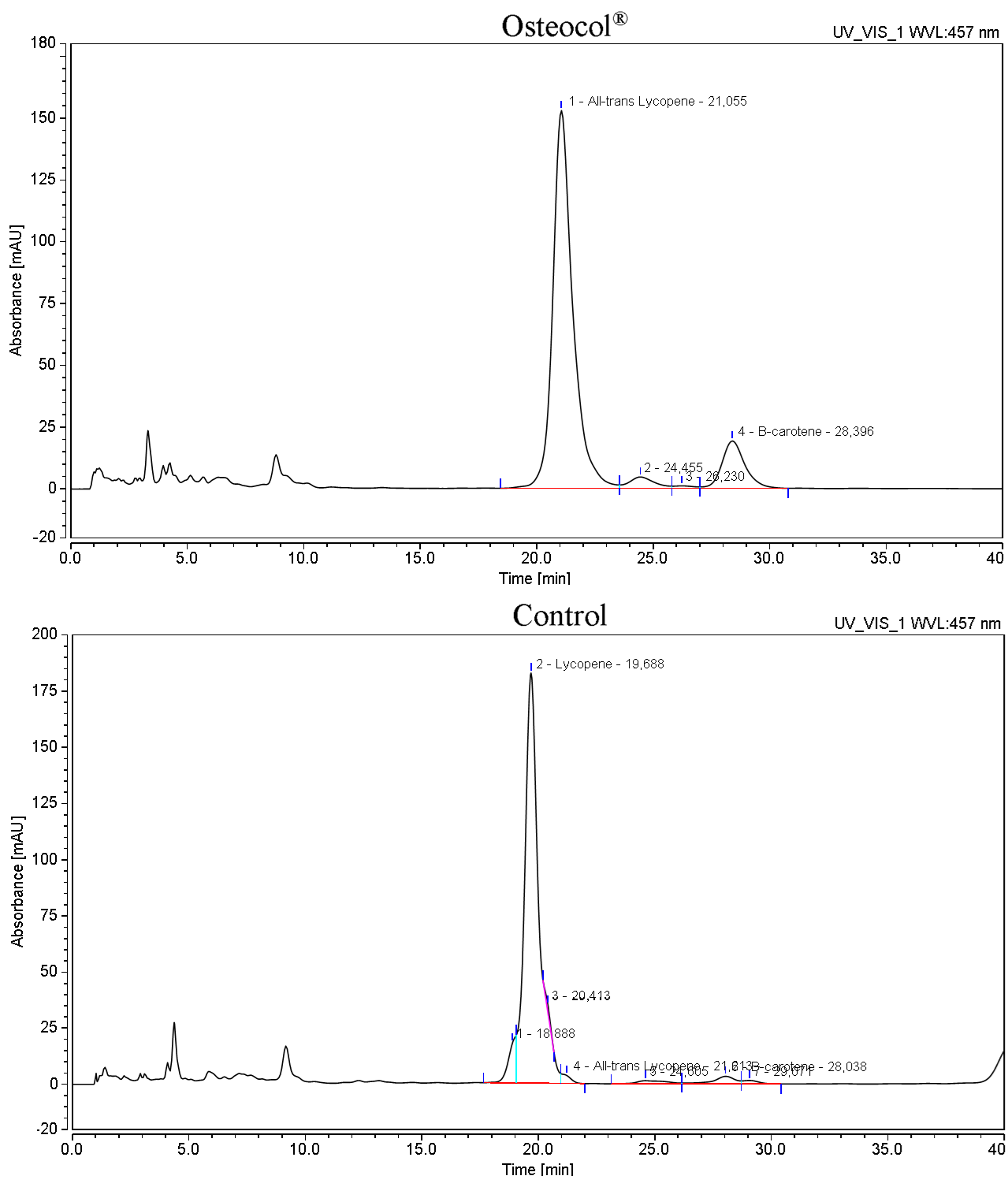
 **Supplemental Figure 1.** HPLC analyses of carotenoids extracted from tomato sauces. Retention times of lycopene in cis/trans isoform, all trans-lycopene and β-carotene were 19.68 min, 21.05 min and 28.39 min, respectively.


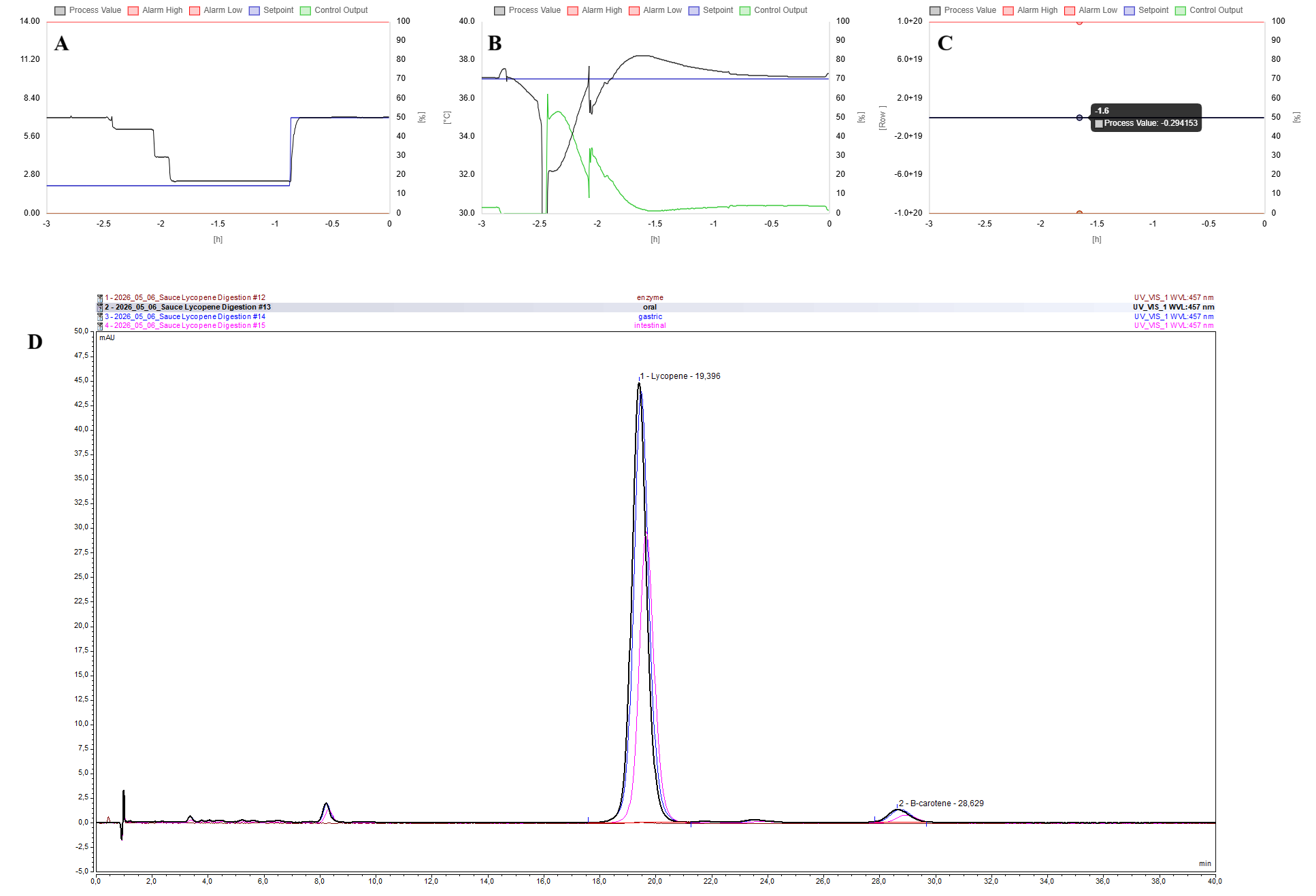


**Supplemental Figure 2.** Carotenoids HPLC analyses in all *in vitro* digestive phase. pH (A), temperature (B) and CO_2_ (C) changes in digestive procedures. HPLC quantification in digested samples (D).


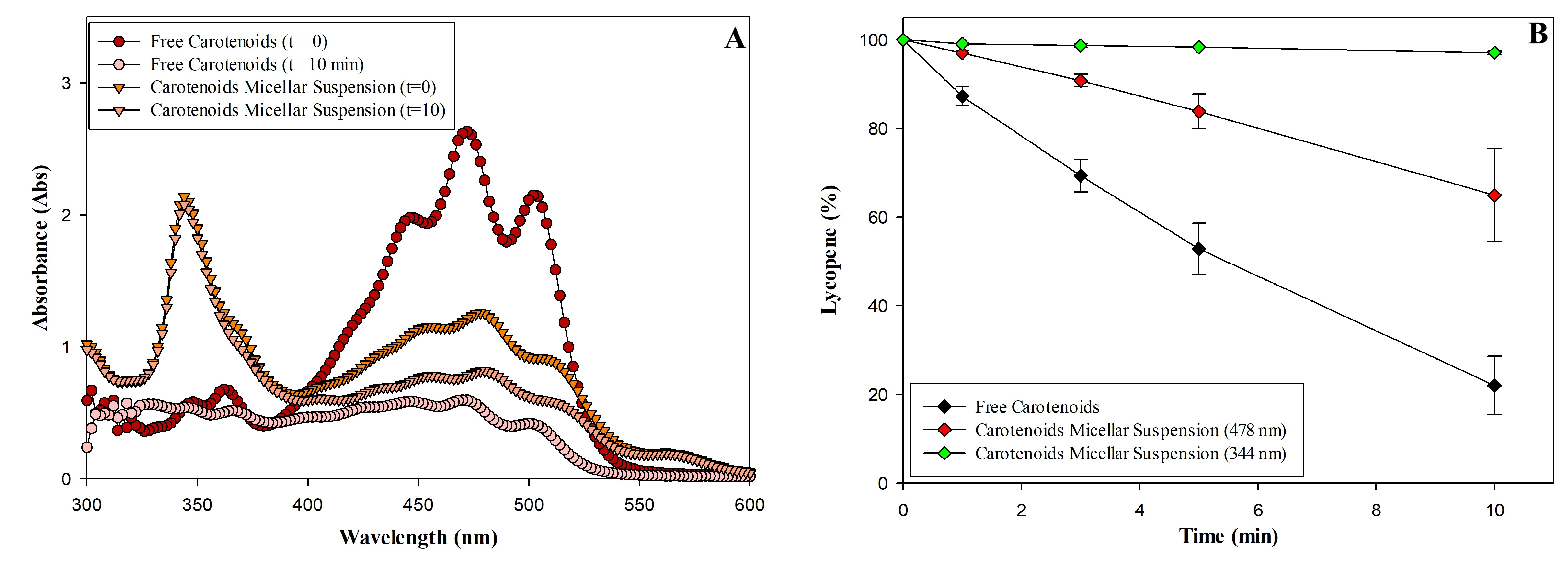


**Supplemental Figure ~~2~~ 3.** Spectrophotometric scansion in UV-Vis wavelength range of free-lycopene solubilized in hexane (Panel A - red and pink lines) and micellar lycopene in water (Panel A - orange and light orange lines) for *in vitro* study. Stability test of aforementioned lycopene samples, respectively in hexane (Panel B – black spots) or in micellar suspension (Panel B – red and green spots), up to 10 minutes exposure to UV radiation.


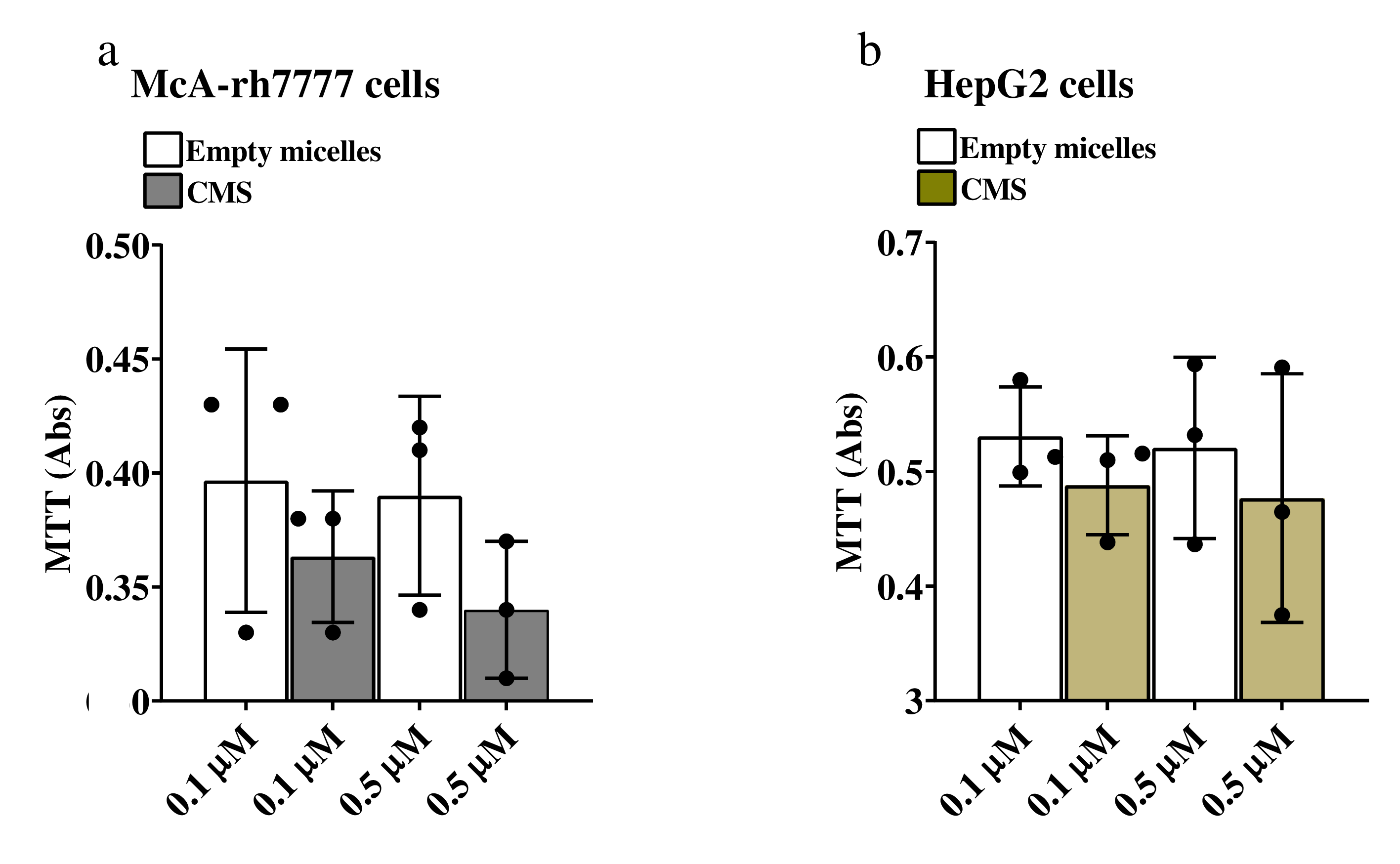


**Supplemental Figure ~~3~~ 4.** CMS does not influence cell viability. (A) Cell viability on McA-RH7777 and (B) HepG2 cells by MTT assay. Data are represented as mean ± SD of three independent experiments.


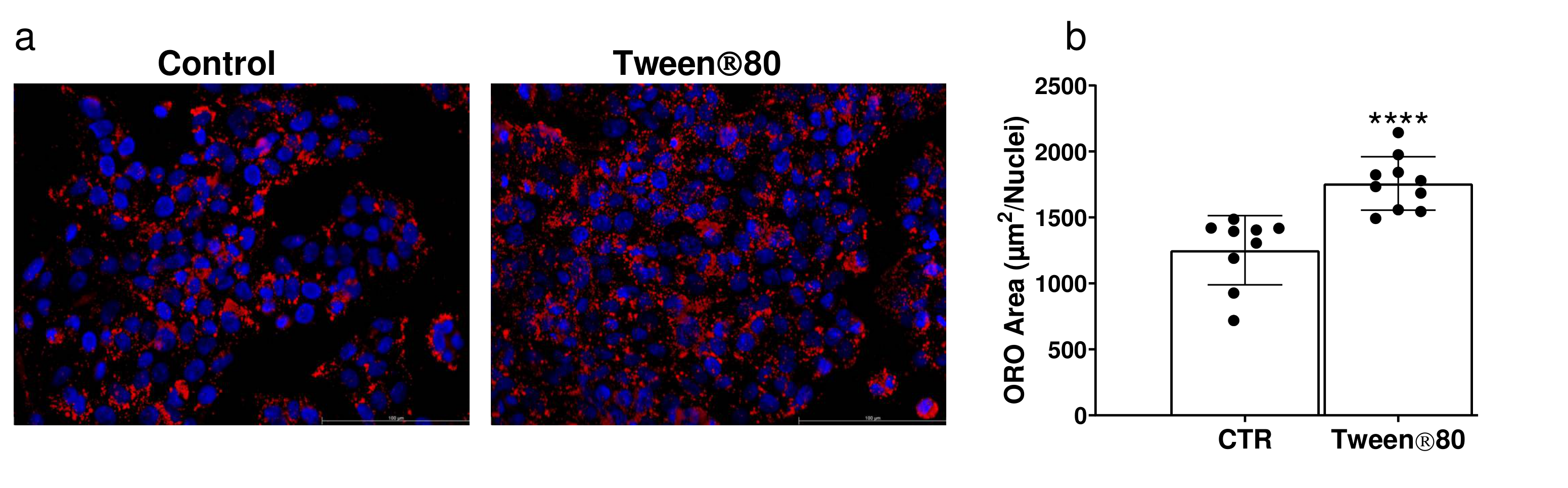
Supplementary Figure 5: **Empy micelles (Tween 80®) increases neutral lipids content in HepG2 cells.** (A) Intracellular lipid content was measured by Oil red-O staining and (B) ORO area quantified by Image J. Data are represented as mean ± SD and p-values are calculated by Student’s t-test (**** p<0.0001).


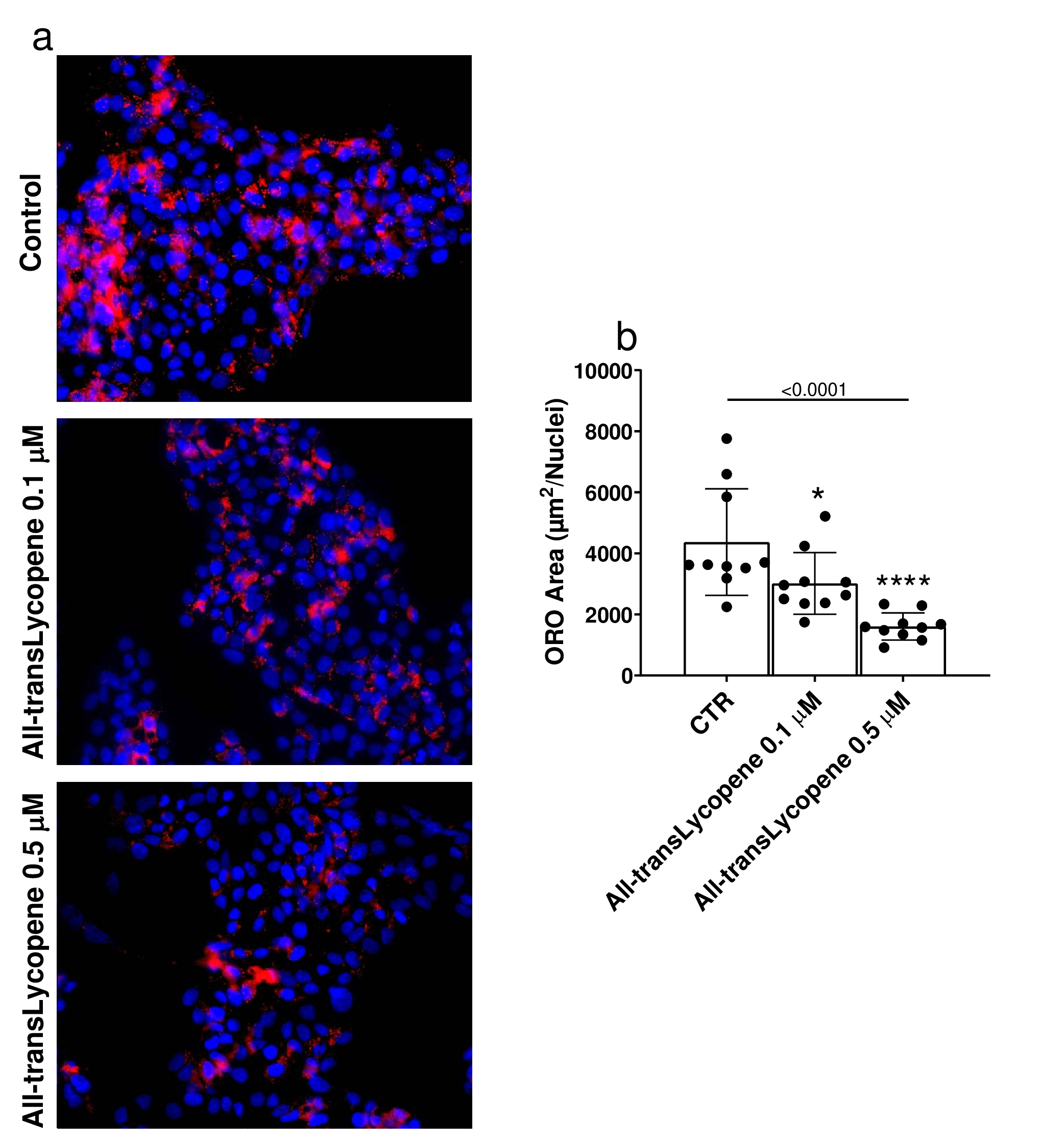


Supplementary Figure 6: ***All-trans* Lycopene reduces neutral lipids content in HepG2 cells.** (A) Intracellular lipid content was measured by Oil red-O staining and (B) ORO area quantified by Image J. Data are represented as mean ± SD and p-values are calculated by Student’s t-test (*p<0.05; **** p<0.0001) and Linear regression.
